# Cell size sets the diameter of the budding yeast contractile ring

**DOI:** 10.1038/s41467-020-16764-x

**Published:** 2020-06-11

**Authors:** I. V. Kukhtevich, N. Lohrberg, F. Padovani, R. Schneider, K. M. Schmoller

**Affiliations:** 1 Institute of Functional Epigenetics, Helmholtz Zentrum München, 85764 Neuherberg, Germany; 2 German Center for Diabetes Research (DZD), 85764 Neuherberg, Germany

**Keywords:** Biophysics, Cell biology, Cell division, Cell growth, Cell polarity

## Abstract

The formation and maintenance of subcellular structures and organelles with a well-defined size is a key requirement for cell function, yet our understanding of the underlying size control mechanisms is limited. While budding yeast cell polarization and subsequent assembly of a septin ring at the site of bud formation has been successfully used as a model for biological self-assembly processes, the mechanisms that set the size of the septin ring at the bud neck are unknown. Here, we use live-cell imaging and genetic manipulation of cell volume to show that the septin ring diameter increases with cell volume. This cell-volume-dependence largely accounts for modulations of ring size due to changes in ploidy and genetic manipulation of cell polarization. Our findings suggest that the ring diameter is set through the dynamic interplay of septin recruitment and Cdc42 polarization, establishing it as a model for size homeostasis of self-assembling organelles.

## Abstract (web summary)

Budding yeast cell polarization is known to self-assemble, but it is still not clear what controls the size of the resulting septin ring. Here the authors show that the septin ring diameter is set by cell volume, ensuring that larger cells have larger rings.

## Introduction

One of the defining properties of living systems is the ability to build structures with precise spatiotemporal organization through self-assembly processes that occur at multiple length and time scales. While self-organization is ubiquitous in biology, focusing experimental and theoretical studies on a small number of well-defined and easily accessible model processes facilitates a deep mechanistic understanding of potentially general regulatory principles. One such a model system is the formation of the bud site in the budding yeast *Saccharomyces cerevisiae*. Importantly, bud site formation combines several fundamental aspects common to many cellular processes^1,2^. First, the selection of one, and only one, bud site requires a symmetry break leading to polarization of the cell despite its high geometric symmetry. Second, once the cell is polarized, a bud neck with a distinct size has to be formed at the selected site. Third, this process needs to be controllable; in particular, cells need to be able to control the timing of bud site formation to coordinate it with other cell cycle events^3,4^.

**Fig. 1:**
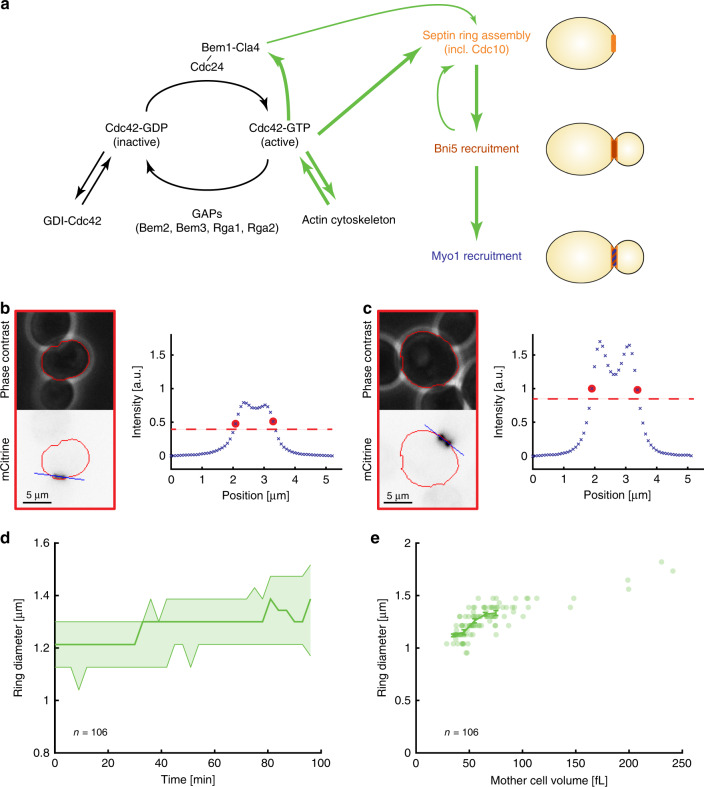
Septin ring diameter increases with cell volume. **a** Illustration of the regulatory pathways controlling cell polarization and downstream ring assembly at the bud neck. Representative live-cell microscopy images (phase contrast (top) and Cdc10-mCitrine fluorescence (bottom)) of a small (**b**) and big (**c**) cell 15 min before the disappearance of the septin ring. Red outline shows automated cell segmentation based on phase contrast used to estimate cell volume. Blue shows the automatically determined line parallel to the ring used to estimate ring diameter. The corresponding fluorescence intensity profiles after applying gliding average and background subtraction are shown for both cells. Red circles show positions used to estimate septin ring diameter, dashed red lines denote half maximum. **d** The median ring diameter (thick line) as quantified from Cdc10-mCitrine fluorescence signal is shown as a function of the time since the first frame where the ring was detected for analysis. Error bars show 25 and 75 percentiles. Data for a total of 106 cells pooled from three independent experiments are shown for the time range during which a ring was detected for at least 15 cells. **e** For each cell (*n* = 106), the median ring diameter during the time when the ring is detected is shown as a function of the median cell volume during that time. Raw data are shown in Supplementary Fig. 1f. Error bars denote means and standard errors. Cells were grown on SCGE. Source data are provided as a Source Data file.

Significant progress has already been made in understanding the regulatory mechanisms that control bud site selection. Briefly, cell polarization is centered around the localization of the GTPase Cdc42 at the membrane, and its cycling between an active GTP- and an inactive GDP-bound-state, promoted by GTPase-activating proteins (GAPs) and the guanine nucleotide-exchange factor Cdc24 (Fig. 1a, for a detailed review see^1^). Cdc42-dependent recruitment of Cdc24^5–7^, as well as Cdc42-dependent actomyosin-driven transport of Cdc42 to the membrane^8^, establishes positive feedback loops that, together with the guanine nucleotide dissociation inhibitor-dependent selective extraction of Cdc42-GDP from the membrane^9,10^, result in the formation of a single cluster of active Cdc42.

On top of being the origin of cell polarization, the Cdc42 system links cell polarization to upstream cell cycle regulation^3,4^, and downstream formation of the bud neck. In particular, Cdc42-GTP interacts with septins, a conserved group of GTP-binding proteins that assemble into heterooligomeric structures. In budding yeast, interplay of Cdc42-GTP with the septins Cdc3, Cdc10, Cdc11, Cdc12, and Shs1^1^ results in the formation of a septin ring at the site of Cdc42 polarization^11^. This septin ring then forms the basis for the subsequent recruitment of the contractile actomyosin ring via the septin-associated protein Bni5^12,13^. Importantly, while we here refer to the septin structure as the septin ring throughout its presence at the bud neck, it is, in fact, a dynamic structure: from the initial ring before bud formation it transitions into a septin hourglass with septin filaments aligned along the mother-bud axis during bud growth, and finally into a double ring of circumferential filaments during cytokinesis^14^.

In contrast to the progress made in understanding the budding yeast Cdc42-based polarization, the mechanisms that determine the size of the resulting structures at the bud neck are still unclear. While in symmetrically dividing cells with simple geometry the diameter of the contractile ring is a given geometric constraint^15^, it is unknown what determines the diameter of the septin structure at the mother-bud neck in budding yeast. The fact that septins assemble into ring-like structures in vitro^16^, together with the early appearance of septins at the site designated for bud formation, might suggest that also in vivo the septin ring diameter is an intrinsic property of septin assembly that could also determine the diameter of the contractile actomyosin ring, which is assembled downstream of septins. However, several recent observations contradict this hypothesis. First, the diameter of the budding yeast contractile ring has been shown to monotonically increase with cell ploidy^17^. Second, the deletion of GAPs promoting Cdc42-GTP hydrolysis results in an increased Cdc42-cluster size at the site designated for bud formation, going along with an increased septin ring diameter^11^. Together with the observation that in fission yeast the size of the Cdc42-GTP cluster is set by the local radius of cell curvature^18^—and therefore increases with cell size—this suggests a role of Cdc42-GTP cluster size homeostasis in controlling the septin ring diameter. Finally, analysis of deletion mutants with increased bud-neck width revealed a potentially causal correlation of cell size and neck width^19^.

Here, we combine live-cell imaging with genetic modulation of cell volume to show that the diameters of the budding yeast septin and actomyosin rings increase with increasing cell volume. We observe an increase of ring diameter with cell volume using different types of genetic cell volume manipulations and, with a weaker dependence, during replicative aging. In addition, the observed cell-volume dependence is independent of whether we base ring diameter measurements on fluorescently tagged septin Cdc10, Bni5, or Myo1, and is robust toward changes in cell ploidy. While also the size of the initial Cdc42-GTP cluster increases with cell volume, we find that—in contrast to fission yeast—local cell curvature is not the major predictor of Cdc42-GTP cluster size. Interestingly, Cdc42-GTP cluster size and septin ring diameter can be decoupled through the deletion of the formin *BNI1*, suggesting that septin ring diameter is established during the complex interplay of Cdc42-GTP and septin recruitment. Our results highlight the potential of self-assembly processes to control not only the formation but also the size homeostasis of cellular structures.

## Results

### Septin ring diameter increases with cell volume

To determine how the septin ring diameter at the mother-bud neck depends on cell volume, we tagged the endogenous copy of the septin *CDC10* with the fluorescent protein *mCitrine* and used a microfluidics-based live-cell microscopy setup to image haploid cells growing on synthetic complete medium with 2% glycerol 1% ethanol as carbon source (SCGE) for several hours. We chose 2% glycerol 1% ethanol as the carbon source rather than glucose to increase the accessible cell volume range. Using semi-automated software, we segmented cells based on phase contrast^20^, and septin rings based on Cdc10-mCitrine fluorescence (Fig. 1b, c, see “Methods” for details). Briefly, we used a manually determined threshold to obtain a binary image, which allowed us to automatically track the position and orientation of the ring over time. Based on this information, we then obtained a brightness profile from the original fluorescence image parallel to the ring and defined ring diameter as the full width at half maximum of the fluorescence intensity line profile. Importantly, the fact that in the microfluidic device cells are in a quasi-2D environment and typically align such that the bud is in the same focal plane as the mother cell allows us to extract the ring diameter from single epifluorescence images. Indeed, the measurements from single epifluorescence images are quantitatively consistent with control measurements of the diameter based on confocal z-stacks (Supplementary Fig. 1a). Moreover, we find that the measured ring diameter is not sensitive to the illumination intensity used (Supplementary Fig. 1b). To validate our estimation of cell volume from phase contrast images, we compared it with fluorescence-based 3D reconstruction using confocal z-stacks, as well as to total fluorescence intensity of mKate2 expressed from an *ACT1* promoter, which is an alternative proxy for cell size^21,22^ (Supplementary Fig. 1c–e). In both cases, we found a strong correlation.

Consistent with a recent study^19^, we observe a slight increase of septin ring diameter during the cell cycle (Fig. 1d and Supplementary Fig. 2a). To address whether the ring diameter depends on cell volume, we calculated the median diameter and median mother cell volume (not including the bud) during the time in which the ring was detected by our segmentation approach. Here, using the median over several time points minimizes the experimental error caused by errors in cell segmentation or ring detection at single time points. As shown in Fig. 1e, we find a clear positive correlation of ring diameter with mother cell volume (*p* < 10^−22^). Moreover, the data suggest that this increase is not directly proportional: a doubling of cell volume from 50 to 100 fL results in an increase of ring diameter by <50%, suggesting that ring diameter might increase in proportion to the “cell diameter”, i.e., the linear dimension of the cell. However, cell-to-cell variability of the measured ring diameter at a given measured cell volume, either due to measurement error or actual variability, combined with the narrow cell volume distribution of steady-state wild-type cells complicates quantitative determination of the underlying cell-volume dependence based only on the analysis of the limited range of cell volumes in wild-type cells.

**Fig. 2:**
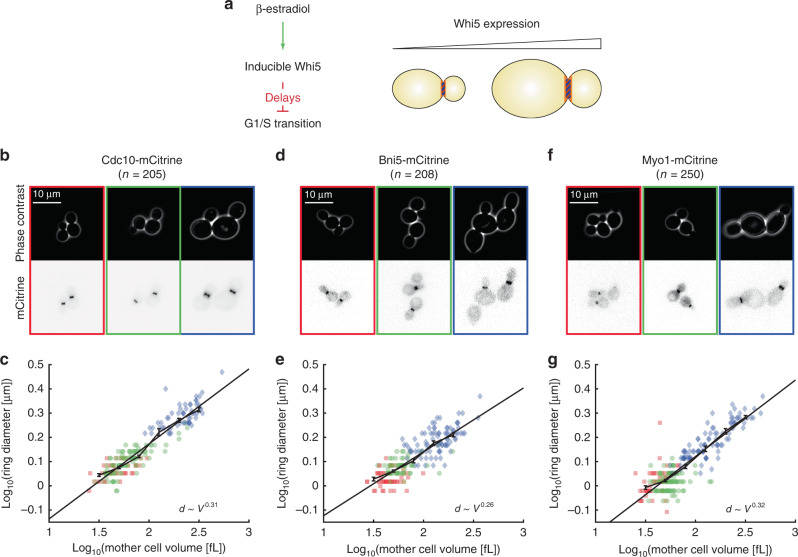
Contractile ring diameter scales with cell volume for cells grown on SCGE. **a** Strains carrying β-estradiol-inducible *WHI5* were used to manipulate cell volume. Whi5 inhibits the G1/S transition, and continuous Whi5 overexpression therefore results in larger steady-state cell volumes. **b**–**g** Using this system, we obtained steady-state cell populations with smaller (0 nM β-estradiol: red, squares) and larger (30 nM β-estradiol: blue, diamonds) volumes compared with wild type (green, circles). The ring proteins Cdc10 (**b**, **c**), Bni5 (**d**, **e**) and Myo1 (**f**, **g**) were tagged with mCitrine in separate strains to visualize the ring and measure the ring diameter at different cell cycle stages. **b**, **d**, **f** For each tagged protein, representative live-cell microscopy images for each condition (left: 0 nM β-estradiol; middle: wild type; right: 30 nM β-estradiol) are shown (phase contrast (top) and mCitrine fluorescence (bottom)). **c**, **e**, **g** For each cell, the median ring diameter during the time when the ring is detected is shown as a function of the median cell volume during that time (**c** 205 cells pooled from six independent experiments, **e** 208 cells pooled from four independent experiments, **g** 250 cells pooled from five independent experiments; linear plots are shown in Supplementary Fig. 2d–f). Data from different conditions are pooled and linear fits to the double-logarithmic data as well as binned means with standard error are shown for each tagged protein. Cells were grown on SCGE. Source data are provided as a Source Data file.

To extend the experimentally accessible range of cell volumes, we constructed a strain that carries in addition to fluorescently tagged *CDC10* a β-estradiol-inducible *WHI5* allele, replacing the endogenous copy (previously described in ref. ^23^). Whi5 is an inhibitor of the transcription factor SBF^23–25^, which controls a large set of genes required for S-phase entry^26^ (Fig. 2a). By controlling cell cycle entry in a size-dependent manner^23^, Whi5 acts as a cell size regulator. Thus, by tuning Whi5 concentration using the artificial controllable promoter^27^, we can strongly alter steady-state cell volume without major effects on population doubling time. In the absence of β-estradiol, the cells are slightly smaller than the wild type, as expected for *whi5* deletion mutants, whereas addition of 30 nM β-estradiol results in steady-state populations with ~3-fold increase in average cell volume (Fig. 2b).

Using the strain with inducible Whi5 as described above, we were able to obtain budding cells with mother volumes ranging from ~30 to 300 fL (Fig. 2b). Increasing cell volume through overexpression of Whi5 resulted in a pronounced increase of the septin ring diameter (Fig. 2c and Supplementary Fig. 2d). Because the ring diameter in wild-type cells seems to follow the same cell-volume dependence observed for cells carrying inducible Whi5—either in the absence or presence of β-estradiol inducing Whi5 expression—we decided to pool the single-cell data obtained for both strains to quantify the volume dependence over the whole range of cell volumes.

If plotted in a double-logarithmic fashion, the data are consistent with a linear increase, suggesting that the dependence of the ring diameter on cell volume can be well described by a power law $d\sim V^{0.31}$. Indeed, comparison with simple alternative models such as a linear ($d = 1.03 + 3.8 \, \times 10^{ - 3} \times V$) or logarithmic model $\left( {d = - 0.67 + 1.1 \times {\mathrm{log}}_{10}(V)} \right)$ reveals that the power law model minimizes the squared residuals best. Remarkably, the observed exponent is close to 1/3 (0.29–0.33, based on 95% confidence intervals obtained from 50,000 bootstraps of the linear fit), which would be expected for spherical cells in which the septin ring diameter increases with cell diameter. Accordingly, we find that the septin ring diameter increases approximately in direct proportion to the length of the cell measured along its major axis (Supplementary Fig. 2g).

### Ring diameter increases with cell volume throughout budding

Next, we investigated whether the dependence of ring diameter on cell volume holds similarly true at later stages of the cell cycle, where different proteins are present at the bud-neck ring structure. As representative proteins that appear at the ring at later stages of the cell cycle, we chose Bni5 and Myo1. Myo1 is the heavy chain of the budding yeast type II myosin, which is a key component of the contractile actomyosin ring, and Bni5 plays an important role in the assembly of the contractile ring by binding to the septin ring and then recruiting Myo1 to the bud neck^12,13^ (Fig. 1a). We repeated the experiments described above with strains where we fluorescently tagged the ring proteins Bni5 and Myo1, respectively, instead of the septin Cdc10. First, we ensured that also for Bni5 and Myo1 the ring diameter as estimated from the respective fluorescence signal does not change majorly during the period in which the respective protein is localized at the bud neck (Supplementary Fig. 2b, c). Since we were able to confirm this, we again used the median ring diameter during that period as a single parameter to quantify its dependence on cell volume. As shown in Fig. 2d–g and Supplementary Fig. 2e, f, the ring diameters estimated from either Bni5 or Myo1 show sublinear cell-volume dependencies similar to that observed for Cdc10. Again, the power laws obtained by linear fits to the double-logarithmic data (95% confidence intervals: 0.24–0.29 for Bni5; 0.29–0.33 for Myo1) imply that the ring diameter is roughly proportional to the linear dimension of the cell (Supplementary Fig. 2h, i). Moreover, the fact that we observe similar cell-volume dependences for ring diameters estimated from fluorescently tagged Cdc10, Bni5, and Myo1 suggests that the cell-volume-dependence is set at an early stage, potentially septin ring assembly, and then maintained from bud emergence to mitosis. Interestingly, for all three proteins, we find that the total fluorescence intensity in the ring after subtraction of background fluorescence increases roughly in proportion to the ring diameter (Supplementary Fig. 2j–o). Consistent with previous work in early *C. elegans* embryos^15^ and the fungus *N. crassa*^28^, this suggests that while ring diameter increases with cell volume, the local structure of the ring is largely maintained.

### Ring diameter increases with cell volume on glucose media

**Fig. 3:**
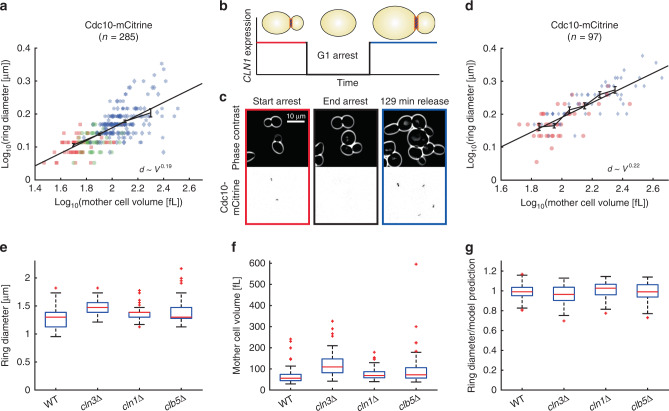
Ring diameter increases with cell volume for cells grown in SCD and is not sensitive to CDK activity. **a** Septin ring diameter based on Cdc10-mCitrine fluorescence as a function of cell volume. Cell volume was controlled through β-estradiol-dependent expression of Whi5 (red squares: 0 nM; green circles: wild type; blue: 60 nM (diamonds), 90 nM (pentagrams), 120 nM (hexagrams)). For each cell, the median ring diameter during the time when the ring is detected is shown as a function of the median cell volume during that time. Data from different conditions are pooled (285 cells from five independent experiments) and a linear fit to the double-logarithmic data as well as binned means with standard error is shown. **b** A *cln1/2/3Δ* strain carrying a β-estradiol-inducible *CLN1* allele and *CDC10-mCitrine* was used to measure septin ring diameter as a function of cell volume. Ring diameter was measured for cells growing in the presence of β-estradiol, or cells released from G1 arrest after 2.5 h of β-estradiol withdrawal. **c** Representative live-cell microscopy images (phase contrast (top) and mCitrine fluorescence (bottom)). **d** For each cell, the median ring diameter is shown as a function of the median cell volume. Pre-arrest (red circles) and post-G1-arrest data (blue diamonds) were pooled (97 cells from three independent experiments), and a linear fit to the double-logarithmic data as well as binned means with standard errors is shown. Cells were grown on SCD. **e**–**g** Cell volume and septin ring diameter based on Cdc10-mCitrine fluorescence were measured in haploid cyclin deletion mutants *cln3Δ* (64 cells pooled from two independent experiments), *cln1Δ* (97 cells pooled from two independent experiments), and *clb5Δ* (52 cells pooled from two independent experiments) and compared with wild-type haploids (106 cells pooled from three independent experiments). Boxplots show distributions of median septin ring diameters (**e**) and median cell volume (**f**). **g** For each cell, we compare the measured ring diameter to the value expected for wild-type cells with the same mother cell volume based on the power law fit obtained for wild-type and inducible-Whi5 cells shown in Fig. 2c. Boxplots depict the distribution of the ratios of measured and expected ring diameters. Cells were grown on SCGE. Boxplots depict medians, 25 and 75 percentiles. Whiskers denote extreme values still within 1.5 interquartile ranges, red symbols denote outliers. Source data are provided as a Source Data file.

Next, we asked whether the observed scaling behavior is robust toward changes in the growth conditions. We therefore repeated the experiment described above for cells with fluorescently tagged Cdc10 grown in synthetic complete 2% glucose medium (SCD). To obtain a similar range of cell volumes compared with cells grown on SCGE, we used several different concentrations of β-estradiol (60, 90, and 120 nM). Consistent with the results obtained in Fig. 2, we found that the septin ring diameter increases with cell volume (Fig. 3a). However, we observe a lower power law exponent ($d\sim V^{0.19}$, 95% confidence intervals: 0.16–0.22), suggesting that growth condition modulates the exact scaling behavior.

### Ring diameter increases with cell volume after G1 arrest

While Whi5 overexpression provides a physiological way of manipulating steady-state cell volume, it could be possible that the observed dependence of ring diameter on cell volume originates from a Whi5-specific effect. We therefore decided to take an alternative approach of manipulating cell volume by temporarily arresting cells in the G1 phase. Specifically, we fluorescently tagged Cdc10 in a previously described strain in which all three G1 cyclins *cln1/2/3* are deleted, and which is kept alive through expression of *CLN1* from a β-estradiol-inducible promoter^29^ (Fig. 3b). We grew and imaged cells in SCD in the presence of β-estradiol in a microfluidic device, and after 2 h switched to medium without β-estradiol. As shown in Fig. 3c, this resulted in a G1 arrest during which arrested cells kept growing. After 2.5 h, we switched back to medium containing β-estradiol, which for many cells resulted in a release from G1 arrest. We then analyzed septin ring diameter and cell volume for cells in which the septin ring appeared either before or after the arrest period. As shown in Fig. 3d, the G1 arrest significantly increased the range of mother cell volumes observed. Again, we find that the septin ring diameter increases with cell volume ($d\sim V^{0.22}$, 95% confidence intervals: 0.19–0.26), demonstrating that the scaling of ring diameter with cell volume we observed is comparable between different cell size manipulation strategies and not simply due to Whi5-specific effects.

### Septin ring diameter is not sensitive to cyclin deletions

Activity of the cell cycle kinase Cdk1 has recently been suggested to play a role not only in the initial polarization of the cell but also in the recruitment of septins to the presumptive bud site^4,30^. Since cell size control is intricately linked to the cell cycle, this raises the question of whether CDK activity might play a direct role in controlling septin ring diameter. We therefore measured septin ring diameter based on Cdc10-mCitrine fluorescence and cell volume in strains growing on SCGE in which we deleted the G1 cyclin *CLN3*, the G1/S cyclin *CLN1*, or the S-phase cyclin *CLB5*, respectively. We found that *cln3Δ* cells build larger septin rings (Fig. 3e), which is consistent with their larger cell volume (Fig. 3f). To investigate whether the increased cell volume quantitatively accounts for the larger septin ring, we made use of the fact that for wild-type cells we have measured the dependence of septin ring diameter as a function of cell volume over a wide range of volumes using the inducible-Whi5 system (Fig. 2c). This allowed us to compare for each mutant cell the measured ring diameter with the diameter expected for a wild-type cell of similar volume. Specifically, we calculated for each cell the relative deviation from the power law fit obtained in Fig. 2c (Fig. 3g). We find for all three cyclin deletions that the deviation from the prediction based on cell volume is small and comparable to the 95% confidence interval of the power law fit (±1–3%, depending on cell volume). Thus, septin ring diameter is not sensitive to the reduction of CDK activity caused by the deletion of individual G1-S cyclins.

### Septin ring diameter increases with replicative age

**Fig. 4:**
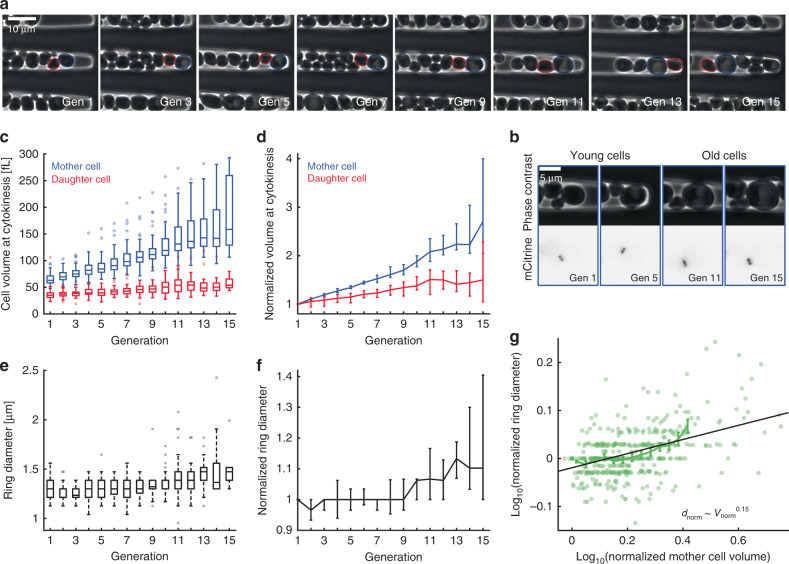
Septin ring diameter increases with age-dependent mother cell volume. **a** Newborn cells trapped in a cavity were imaged for multiple generations. Phase contrast images are shown for a representative mother cell at the time points of cytokinesis from first to fifteenth division. Mother and daughter segmentations are shown in blue and red, generation numbers are denoted in the images. **b** Zooms of phase contrast (top) and mCitrine fluorescence (bottom) images corresponding to the aging cell shown in **a** are shown for time points where the septin ring is visible. **c** Mother and daughter cell volumes at the time point of cytokinesis are shown as a function of generation number. **d** Cell volumes normalized on the volume at the first division are shown as a function of generation number. **e** Median septin ring diameter during the time when the ring is detected based on Cdc10-mCitrine fluorescence is shown as a function of generation number. **f** Septin ring diameter normalized on the diameter during the first bud event is shown as a function of generation number. **g** For each cell, the normalized ring diameter is shown as a function of the corresponding normalized mother cell volume. A linear fit to the double-logarithmic data as well as binned means with standard error is shown. Boxplots in **c**, **e** show medians and 25 and 75 percentiles. Whiskers denote extreme values still within 1.5 interquartile ranges, symbols denote outliers. Error bars in **d**, **f** denote 95% confidence intervals as determined from 50,000 bootstrap samples. Cells were grown on SCD. Data are based on 44 cells from four independent experiments tracked over at least nine generations. Source data are provided as a Source Data file.

So far, we have shown that different genetic manipulations of cell volume result in changes of septin ring diameter. This analysis was mostly based on young cells, which dominate in exponentially proliferating populations. However, one important physiological process resulting in an increased cell volume is the replicative aging of budding yeast mother cells. In fact, if cells become too big, the size increase itself impairs cell function and thereby contributes to aging^31^. We therefore asked whether the age-dependent increase of mother cell volume also goes along with an increase in septin ring diameter. We grew cells with mCitrine-tagged Cdc10 in SCD in a recently described microfluidic device that allows imaging mother cells trapped in a cavity over many generations (Fig. 4a, b)^32^. As expected, we find that mother cell volume at cytokinesis, and to a lesser extent the volume of the newborn daughter cells, increases continuously with generation number (Fig. 4c, d). While the diameter of the septin ring seems to be rather constant during the first cell cycles, it noticeably increases for mother cells older than ten generations (Fig. 4e, f). To obtain more direct insights into the dependence of septin ring diameter on age-dependent cell volume increase, we asked how the relative increase of ring diameter compared with the first budding event depends on the relative increase of cell volume. As shown in Fig. 4g, we find that the increase of septin ring diameter is correlated with the increased cell volume (*p* < 10^−24^). However, the power law exponent obtained from a linear fit to the double-logarithmic data, $d_{{\mathrm{norm}}}\sim V_{{\mathrm{norm}}}^{0.15}$ (95% confidence intervals: 0.12–0.18), suggests that the age-dependent increase of ring diameter with cell volume is weaker than the dependence on cell volume observed for young cells. Together with the observation that during the first ten generations the ring diameter is largely constant, this indicates that during replicative aging a specific regulatory mechanism maintains a constant ring diameter during the first cell cycles despite the increase of mother cell volume.

### Ploidy dependence of ring size is explained by cell volume

**Fig. 5:**
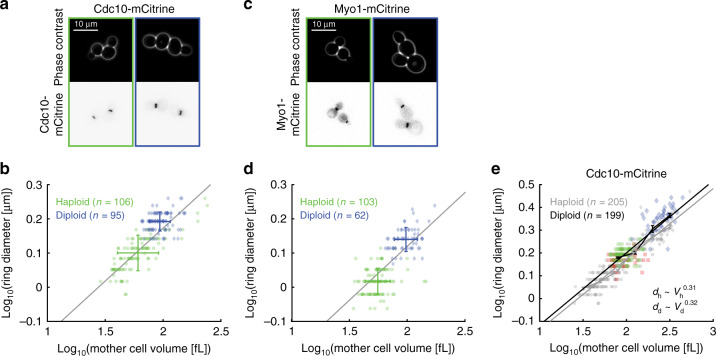
Cell volume explains larger ring diameter in diploids. The ring diameter as obtained from mCitrine-tagged Cdc10 (**a**, **b**) and Myo1 (**c**, **d**) is larger in diploid cells (blue, diamonds) compared with haploids (green, circles). **a**, **c** Representative live-cell microscopy images of haploid (left) and diploid (right) cells are shown (phase contrast (top) and mCitrine fluorescence (bottom)). **b**, **d** For each cell, the median ring diameter during the time when the ring is detected is shown as a function of the median cell volume during that time. Error bars denote mean and standard deviation for haploids (green) and diploids (blue). Gray lines show linear fits obtained in Fig. 2 for haploids of different sizes for comparison. 106 (Cdc10-mCitrine) and 103 (Myo1-mCitrine) haploid cells, each pooled from three independent experiments. 95 (Cdc10-mCitrine) and 62 (Myo1-mCitrine) diploid cells, each pooled from two independent experiments. **e** In addition to wild type (green, circles), a diploid strain carrying a β-estradiol-inducible *WHI5* allele was used to obtain steady-state cell populations of small (0 nM β-estradiol: red, squares) and large (60 nM β-estradiol: blue, diamonds) volumes. Data for haploid cells from Fig. 2 are shown for comparison (gray symbols). Data for each ploidy are pooled (205 haploid cells pooled from six independent experiments; 199 diploid cells pooled from four independent experiments) and linear fits to the double-logarithmic data as well as binned means with standard errors are shown (haploids in gray, diploids in black). Cells were grown on SCGE. Source data are provided as a Source Data file.

It has been previously shown that the diameter of the contractile ring in budding yeast increases with ploidy^17^. While Pinto et al. already suggested that this increase could be due to the increase of cell volume with ploidy^33^, it remains unclear to what extent ploidy itself plays a direct role in controlling ring diameter. To address this question, we measured ring diameter and cell volume in diploid strains with mCitrine-tagged Cdc10 and Myo1, respectively, grown in SCGE medium (Fig. 5a–d). As expected, diploid cells are bigger than haploid cells and build larger rings. Remarkably, for both Cdc10- and Myo1-based measurements, the ring diameter in diploids roughly matches the expectation for haploids of similar volume based on the power law scaling determined in Fig. 2. To determine whether the ring diameter in diploid cells indeed follows the same cell-volume dependence, we constructed a diploid strain with mCitrine-tagged Cdc10 that also carries inducible *WHI5*, which provides us with diploid cells spanning a larger range of cell volumes. Using this approach, we find that the cell-volume dependence of diploids closely matches that of haploid cells (Fig. 5e), with a nearly identical power law exponent ($d\sim V^{0.32}$, 95% confidence intervals: 0.29–0.34), only shifted to slightly larger diameters (7%, *p* < 10^−10^). This demonstrates that the larger ring diameter in diploids is largely a consequence of the larger cell volume and does not require additional ploidy-specific regulation.

### Increased volume of GAP mutants largely explains bigger ring

**Fig. 6:**
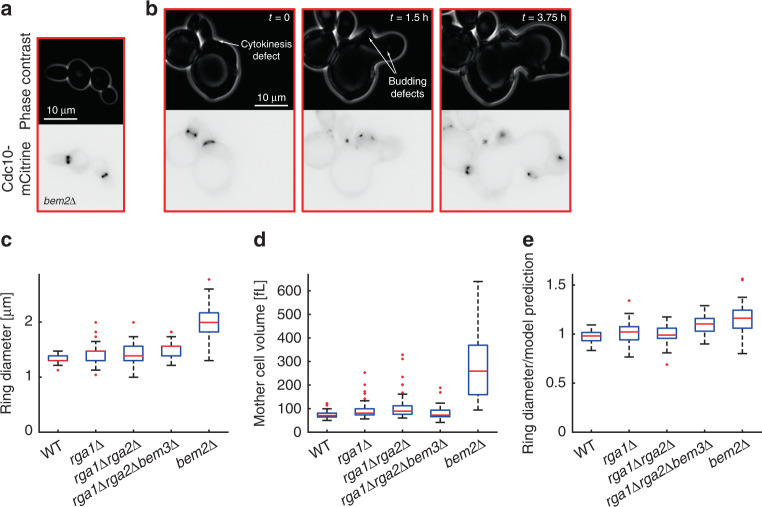
Increased ring diameter in *bem2Δ* cells is largely explained by increased cell volume. Cell volume and septin ring diameter based on Cdc10-mCitrine fluorescence were measured in haploid GAP deletion mutants *rga1Δ* (43 cells pooled from two independent experiments), *rga1Δrga2Δ* (50 cells pooled from two independent experiments), *rga1Δrga2Δbem3Δ* (42 cells pooled from two independent experiments), and *bem2Δ* (42 cells pooled from two independent experiments) and compared with wild-type haploids (52 cells pooled from two independent experiments). Cells with obvious defects in bud formation, cytokinesis, and septin morphology were rejected from the analysis. Live-cell microscopy images of *bem2Δ* cells (phase contrast (top) and mCitrine fluorescence (bottom)) show typical examples of normal ring morphology included in the analysis (**a**), and a timecourse of a typical cell rejected from the analysis due to cytokinesis and budding defects (**b**). **c** Boxplots showing the distribution of septin ring diameters. For each cell, the median ring diameter during the time when the ring is detected is measured. **d** Boxplots of the median cell volume during that time. **e** For each cell, we compare the measured ring diameter to the value expected for wild-type cells with the same mother cell volume based on the power law fit obtained for wild-type and inducible-Whi5 cells grown on SCD shown in Fig. 3a. Boxplots depict the distribution of the ratios of measured and expected ring diameters. Cells were grown on SCD. Boxplots depict medians, and 25 and 75 percentiles. Whiskers denote extreme values still within 1.5 interquartile ranges, red symbols denote outliers. Source data are provided as a Source Data file.

After demonstrating that the increased septin ring diameter in diploid cells is largely explained by the increased cell volume, we asked whether the changes of septin ring diameter in mutant strains with impaired function of GAPs, which were attributed to the decreased GAP activity^11^, may be simply indirect consequences of altered cell volume rather than a mutant-specific effect on septin assembly. Specifically, we measured septin ring diameter based on Cdc10-mCitrine fluorescence and cell volume in strains carrying single (*rga1Δ*, *bem2Δ*) or multiple (*rga1Δrga2Δ*, *rga1Δrga2Δbem3Δ*) deletions of GAP proteins (Fig. 6). Consistent with a previous report^34^, deletion of *BEM2* caused a severe growth defect on SCGE medium, and we therefore decided to perform our analysis on cells growing on SCD. While many *bem2Δ* cells growing on SCD in the microfluidic device exhibit normal cell and septin ring morphologies (Fig. 6a), a subset (~24%)—in particular, the larger cells—exhibited severe defects in bud formation and mitosis (Fig. 6b). For all strains, cells with obvious defects in septin ring morphology were excluded from the analysis. Consistent with Okada et al.^11^, we find that GAP deletions result in increased septin ring diameters, with *bem2Δ* having the strongest effect with a 53% increase compared with wild type (Fig. 6c). At the same time, *bem2Δ* cells are also much larger than wild-type cells^35^ (Fig. 6d). To test whether the increased cell volume accounts for the larger septin ring, we calculated for each cell the relative deviation from the power law fit shown in Fig. 3a (Fig. 6e). For *rga1Δ* and *rga1Δrga2Δ* cells we found that the deviation from the power law fit is in the range of its 95% confidence interval (±1–4%, depending on cell volume). In contrast, while we find that the increased cell volume explains a major part of the increase in septin ring diameter observed in *bem2Δ* cells, both *rga1Δrga2Δbem3Δ* (10%) and *bem2Δ* (16%) cells showed a considerable increase of the septin ring diameter even after accounting for changes in cell volume (Fig. 6e). Additional experiments using a strain carrying inducible Whi5 confirmed that deletion of *BEM2* results in an increase of the septin ring diameter after accounting for cell volume (median increase of 23% across all volumes, Supplementary Fig. 3).

### Ring size is not changed in *CDC42* or *CDC10* hemizygotes

**Fig. 7:**
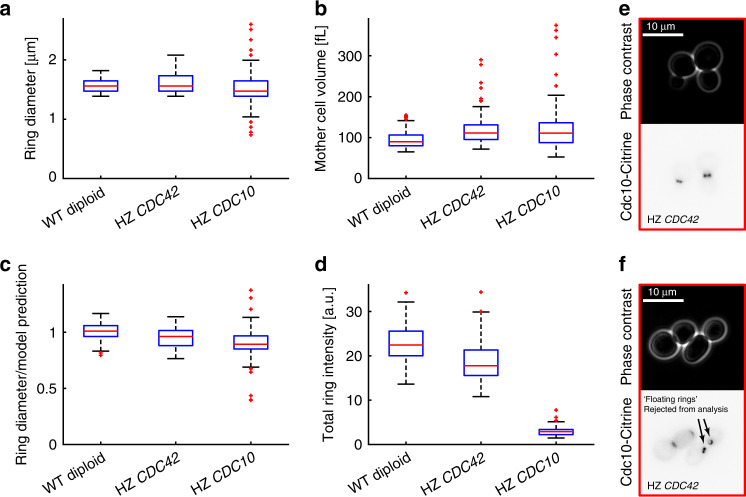
Diploid cells hemizygous for *CDC42* or *CDC10* with normal ring morphology have a similar ring diameter compared with wild-type diploids. Cell volume and septin ring diameter based on Cdc10-mCitrine fluorescence were measured in diploid cells hemizygous for *CDC42* (63 cells pooled from four independent experiments) or *CDC10* (100 cells pooled from two independent experiments) and compared with wild-type diploids (95 cells pooled from two independent experiments). Cells with obvious defects in bud formation, cytokinesis, and septin morphology were rejected from the analysis. **a** Boxplots showing the distribution of septin ring diameters. For each cell, the median ring diameter during the time when the ring is detected is measured. **b** Boxplots of the median cell volume during that time. **c** For each cell, we compare the measured ring diameter to the value expected for wild-type diploid cells with the same mother cell volume based on the power law fit obtained for wild-type and inducible-Whi5 cells grown on SCGE shown in Fig. 5e. Boxplots depict the distribution of the ratios of measured and expected ring diameters. **d** Boxplots of the median total fluorescence intensity (integrated line profile intensity after background subtraction) during the time when the ring is detected. Diploid cells with mCitrine-tagged Cdc10 and hemizygous for *CDC42* often show obvious defects in septin ring morphology, including a frequent occurrence of stable, “free-floating,” fluorescent rings. Live-cell microscopy images (phase contrast (top) and mCitrine fluorescence (bottom)) show typical examples of normal ring morphology included in the analysis (**e**), and cells with obvious defects in septin morphology rejected from the analysis (**f**). Cells were grown on SCGE. Boxplots depict medians, and 25 and 75 percentiles. Whiskers denote extreme values still within 1.5 interquartile ranges, red symbols denote outliers. Source data are provided as a Source Data file.

One way for cells to control the size of organelles is by controlling the abundance of limiting components^36^. Interestingly, the fact that the total Cdc10-mCitrine fluorescence intensity in the septin ring increases with cell volume indicates that the total amount of ring proteins might be involved in controlling the final ring structure. Similarly, the availability of Cdc42 protein might control the size of the polarized Cdc42 cluster, and thereby the downstream septin ring. To test whether the total cellular amount of Cdc42 or Cdc10 may be limiting for the septin ring diameter, we deleted one copy of *CDC42* or *CDC10*, respectively, in diploid cells with mCitrine-tagged *CDC10*. We limited our analysis to the cells that do not show obvious defects in septin ring morphology. We observe a slight reduction of the ring diameter compared with the expectation for wild-type cells, which in the case of *CDC10* (11% reduction) is clearly outside the 95% confidence interval of the power law fit (±1–4%, depending on cell volume) (Fig. 7a–c). However, given the moderate effect, our results suggest that neither the amount of Cdc42 nor of Cdc10 plays a major role in controlling septin ring diameter. Importantly, we observe a dramatic reduction of Cdc10-mCitrine fluorescence intensity in cells hemizygous for *CDC10* to about 13% compared with wild type (Fig. 7d), demonstrating that the amount of Cdc10 is not maintained constant through dosage compensation mechanisms. Cells hemizygous for *CDC42* show a high frequency (51% of cells) of defects in cell or septin ring morphology, including the occurrence of septin rings that are stable and freely floating in the cytoplasm (37% of cells) (Fig. 7e, f), demonstrating that the lack of one *CDC42* allele is not fully compensated. However, in the case of *CDC42*, we cannot fully exclude that the fraction of cells that show normal morphology and were used for the analysis exhibit (partial) dosage compensation of Cdc42 concentration.

### Septin ring diameter is not governed by cell geometry

So far, we have shown that the septin ring diameter increases with cell volume, but whether cell volume is indeed the relevant parameter—or simply correlated with it—is still unclear. In fission yeast, the width of the Cdc42-GTP cluster is determined by the local cell curvature, independent of volume^18^. Moreover, in vitro, septins have been shown to bind to membranes in a curvature-dependent manner^37^. One might therefore speculate that it is in fact local cell curvature that determines septin ring diameter in budding yeast, and that the observed cell-volume dependence follows as an indirect consequence. As a first step to test the impact of cell geometry, we reanalyzed the pooled data on haploid cells grown in SCGE shown in Fig. 2c. We performed a two-variable linear regression (including a constant offset) using the cube root of mother cell volume, $V^{1/3}$, which is a measure for the linear dimension of the cell, and the ratio between the mother cell length along its major axis and the cube root of mother cell volume, $L/V^{1/3}$, which is a measure for how elongated the cell is, to predict septin ring diameter, *d*. While we find the linear dimension of the mother cell to be the major predictor, also its elongation significantly contributes, such that cells of the same volume have larger rings if they are more elongated (*p* < 10^−5^): $d = 0.3V^{1/3} + 0.56L/V^{1/3} - 0.7$. Importantly, we did not observe any correlation between *V*^1/3^ and $L/V^{1/3}$ (*p* > 0.9).

**Fig. 8:**
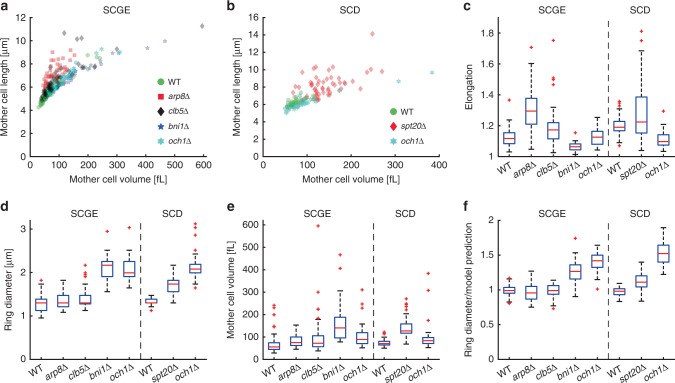
Analysis of the septin ring diameter in geometry mutants. Cell volume and septin ring diameter based on Cdc10-mCitrine fluorescence were measured in haploid geometry mutants grown on SCGE (*arp8Δ* (65 cells pooled from two independent experiments), *clb5Δ* (52 cells pooled from two independent experiments), *bni1Δ* (35 cells pooled from two independent experiments) and *och1Δ* (46 cells pooled from three independent experiments)) or SCD (*spt20Δ* (55 cells pooled from three independent experiments) and *och1Δ* (33 cells pooled from two independent experiments)) and compared with wild-type haploids (106 cells and 52 cells, pooled from three and two independent experiments, respectively). Cell length along the major axis is shown as a function of cell volume for cells grown on SCGE (**a**) or SCD (**b**). **c** Boxplots showing the distribution of cell elongation, which we define as cell length divided by the cube root of volume, normalized such that it is 1 for a sphere. **d** Boxplots showing the distribution of septin ring diameters. For each cell, the median ring diameter during the time when the ring is detected is measured. **e** Boxplots of the median cell volume during that time. **f** For each cell, we compare the measured ring diameter to the value expected for wild-type cells with the same mother cell volume based on the power law fit obtained for wild-type and inducible-Whi5 cells shown in Figs. 2c (SCGE) and 3a (SCD). Boxplots depict the distribution of the ratios of measured and expected ring diameters. Boxplots depict medians, and 25 and 75 percentiles. Whiskers denote extreme values still within 1.5 interquartile ranges, red symbols denote outliers. Source data are provided as a Source Data file.

While this suggests a role of cell geometry in controlling septin ring diameter, it is unclear whether the observed correlation is causal. To test if this is the case, we analyzed five different deletion mutants that modulate the cellular geometry^38^ without strongly affecting septin ring morphology. We grew cells on SCGE medium where possible, but used SCD medium for *spt20Δ* cells, which do not grow on SCGE, and *och1Δ* cells, which we found to only exhibit a round phenotype on SCD (Fig. 8a–c). While the elongated mutants *arp8Δ* and *clb5Δ* show only minor changes of ring diameter after accounting for cell volume, we observed an increased ring diameter in *spt20Δ* cells (11%, Fig. 8d–f). At the same time, ring diameters in the round mutants *bni1Δ* and *och1Δ* are much bigger than expected based on their increased cell volume (28% for *bni1Δ*, 52% for *och1Δ*, Fig. 8d–f). However, deletion of the mannosyltransferase *OCH1* results in an increased ring diameter even on SCGE (42%), where cell geometry is unchanged. In summary, across all mutants, we do not find a consistent effect of cell geometry, and the strong increase of ring diameter in the round *bni1Δ* and *och1Δ* cells is opposite than expected based on our geometry analysis of wild-type cells. Thus, it seems likely that the increased ring diameter in *spt20Δ, bni1Δ*, and *och1Δ* cells is a direct effect of disturbing protein function rather than simply due to the altered cell geometry. Consistent with this view, the formin Bni1, which is involved in actin-mediated transport and septin assembly^30,39^, has recently been shown to affect septin recruitment. Yet using a temperature-sensitive allele, no effects on septin ring size have been observed^30^.

### Cdc42-GTP cluster size increases with cell volume

The finding that *bni1Δ* cells exhibit an increase of septin ring diameter after accounting for cell volume offers an excellent opportunity to gain further insights into the mechanism underlying the regulation of septin ring diameter. Okada et al. reported that across GAP deletion mutants, cells that had larger septin ring diameters also showed larger clusters of active Cdc42 at the site designated for bud formation before septin appearance^11^. Together with the finding that in fission yeast Cdc42-GTP cluster size increases with the local radius of curvature, and thus also cell size^18^, this led us to speculate that septin ring diameter in budding yeast might similarly be determined by the size of the Cdc42-GTP cluster. If septin ring diameter would indeed be causally coupled to Cdc42-GTP cluster size, increasing cell volume or deletion of *BNI1*—both of which result in an increase of the septin ring diameter—should therefore also cause an increase of Cdc42-GTP cluster size.

**Fig. 9:**
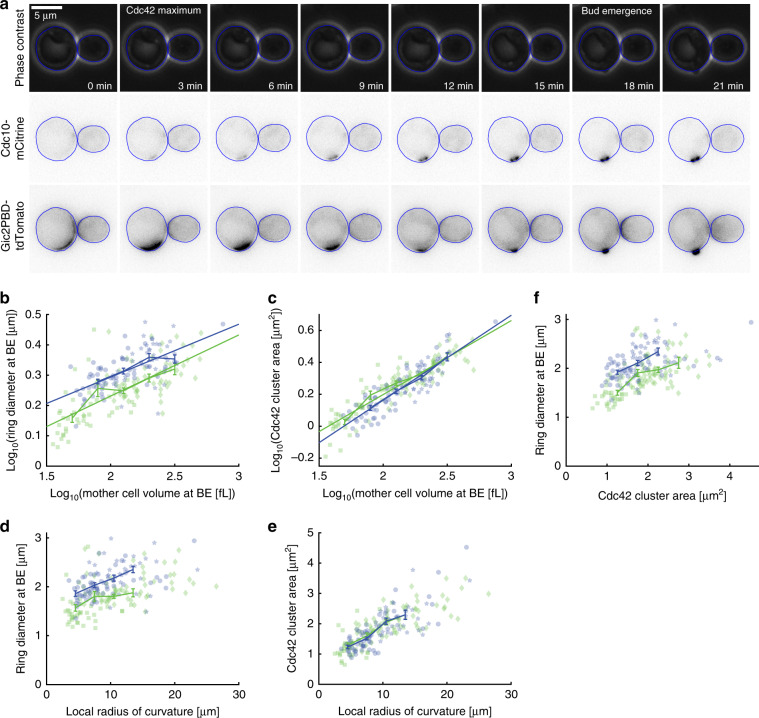
bni1Δ cells have larger septin ring diameters but similar Cdc42-cluster size. **a** Phase contrast and fluorescence microscopy timecourse showing typical dynamics of Cdc42-GTP cluster formation as visualized by Gic2PBD-tdTomato, followed by septin ring formation visualized by Cdc10-mCitrine fluorescence and bud emergence. Cell segmentations are shown in blue. **b**–**e** Cell volume was controlled through β-estradiol-dependent expression of Whi5. Cells that are otherwise wild-type are shown in green (squares: 0 nM β-estradiol, 39 cells; diamonds: 30 nM β-estradiol, 59 cells; each pooled from two independent experiments), *bni1Δ* cells are shown in blue (circles: 0 nM β-estradiol, 57 cells; pentagrams: 30 nM β-estradiol, 38 cells; each pooled from two independent experiments). Septin ring diameter at the time of bud emergence based on Cdc10-mCitrine fluorescence (**b**) and Cdc42-GTP cluster area at peak localization of Cdc42-GTP based on Gic2PBD-tdTomato fluorescence (**c**) is shown as a function of mother cell volume at bud emergence. Septin ring diameter (**d**) and Cdc42-GTP cluster area (**e**) are shown as a function of local radius of cell curvature. **f** Septin ring diameter is shown as a function of Cdc42-cluster area. Error bars show binned means of pooled data with standard errors, solid lines show linear fits to the pooled double-logarithmic data. Cells were grown on SCGE. Source data are provided as a Source Data file.

To test this hypothesis, we constructed strains which in addition to inducible *WHI5* and mCitrine-tagged *CDC10* carry *GIC2PBD-tdTomato*, which was previously used as a reporter for Cdc42-GTP localization^11^. To determine the dependence of Cdc42-GTP cluster size and septin ring diameter on cell volume and local curvature, we grew cells on SCGE in the absence or presence of 30 nM β-estradiol. Consistent with Okada et al., we find that the Gic2PBD signal shows a maximum of localized intensity at the onset of Cdc10 recruitment to the site of ring formation, which is then followed by a transient reduction of Cdc42-GTP intensity at the cluster site (Fig. 9a). For each cell, we measured septin ring diameter, mother cell volume, and the corresponding local radius of cell curvature at the time of bud emergence, as well as the area of the Cdc42-GTP cluster at the time of maximal Cdc42 localization. We find that both septin ring diameter and Cdc42-GTP cluster area increase with cell volume and local radius of curvature (Fig. 9b–e and Supplementary Fig. 4). Consistent with a causal link, septin ring diameter increases as a function of the Cdc42-GTP cluster area (Fig. 9f).

To determine whether cell volume, $V$, or local radius of curvature, $R_C$, is the predictive parameters for septin ring diameter and Cdc42-GTP cluster area, respectively, we performed two-variable linear regressions (including a constant offset), using the cube root of mother cell volume, *V*^1/3^, and the ratio between the local radius of cell curvature and the cube root of mother cell volume at bud emergence, $R_C/V^{1/3}$, as predictive parameters. For the septin ring diameter, *d*, we find that cell volume is the major predictor (*p* < 10^−11^), with a smaller but significant (*p* < 0.012) negative contribution of the local radius of curvature, which could be due to a positive correlation between *V*^1/3^ and $R_C/V^{1/3}$ (*p* < 10^−18^): $d = 0.27\,V^{1/3} - 0.17R_C/V^{1/3} + 0.74$. In contrast, for the area of the Cdc42-GTP cluster, we find that only the cube root of mother cell volume is predictive (*p* < 10^−20^), with the ratio of the local radius of curvature and the cube root of volume adding no significant predictive value (*p* > 0.93). In summary, we find that increasing cell volume indeed results in larger Cdc42-GTP clusters, consistent with a causal link between Cdc42-GTP cluster size and septin ring diameter.

### Deletion of *BNI1* increases ring diameter

To test if deletion of *BNI1*, which results in an increase of septin ring diameter even after accounting for the increased cell volume, also leads to larger Cdc42-GTP clusters, we repeated the experiments described above for *bni1Δ* cells. We find that in *bni1Δ* cells both septin ring diameter at bud emergence (Fig. 9b, d) and Cdc42-GTP cluster area at peak localization (Fig. 9c, e) increase with mother cell volume and local radius of curvature. As in wild-type cells, two-variable linear regression reveals that mother cell volume is the major predictor of septin ring diameter and Cdc42-GTP cluster area. Again, we find that the ratio of the local radius of cell curvature and the cube root of mother cell volume, which is positively correlated with cell volume (*p* < 10^−18^), negatively modulates the increase of septin ring diameter with cell volume (*p* < 0.04) but does not significantly contribute to Cdc42-GTP cluster area (*p* > 0.42). As expected from our previous experiments (Fig. 8), deletion of *BNI1* results in a pronounced increase of septin ring diameter (Fig. 9b, d). Strikingly, for cells with comparable mother cell volume or local radius of curvature, the Cdc42-GTP cluster area at peak localization is unchanged in *bni1Δ* compared with wild-type cells (Fig. 9c, e). This suggests that while Bni1-dependent actin-mediated transport is critical for setting the size of the septin ring, it is not required for correct size homeostasis during the initial stages of Cdc42 polarization. Thus, septin ring diameter is not strictly coupled to the initial Cdc42-cluster size (Fig. 9f), but rather emerges from a complex interplay of Cdc42-GTP and septin recruitment.

## Discussion

In the asymmetrically dividing budding yeast, the Cdc42-based polarization machinery determines the site of bud formation and also establishes the size of the septin ring at the bud neck connecting mother and future daughter cells. Our results demonstrate that the ring diameter is not a fixed property of septin assembly, but instead increases with cell volume. Our finding that the dependence of the ring diameter on cell volume changes depending on the media condition (approximately $d\sim V^{1/3}$ for cells grown on SCGE, and $d\sim V^{1/5}$ for cells grown on SCD) or during replicative aging suggests that ring diameter is tuned according to the cell’s needs. Since also in budding yeast the amount of ring proteins is largely proportional to the ring diameter, the cell-volume dependence of the ring diameter results in a remarkable similarity to cell division in symmetrically dividing cells such as in early *C. elegans* embryos^15^ and the fungus *N. crassa*^28^, in which the initial diameter of the contractile ring is a geometric constraint. However, in contrast to these examples, where the ring diameter is given by the cell diameter at the site of division and therefore trivially increases in direct proportion with cell diameter, the size of the budding yeast contractile ring has to be determined de novo by the self-assembly processes controlling its formation.

Our finding that cell volume largely accounts for the increase of ring diameter observed in diploids or upon deletion of GAP proteins establishes cell volume as a major factor setting ring diameter in budding yeast. It has been previously shown that across GAP mutants, septin ring diameter correlates with Cdc42-GTP cluster size^11^. While the increase of septin ring diameter in GAP mutants is partially a consequence of increased cell volume rather than specific GAP function, we indeed find that also Cdc42-GTP cluster size increases with cell volume. Importantly, in contrast to fission yeast, where cell curvature determines the Cdc42-GTP cluster size^18^, our analysis of geometry mutants and local cell curvature reveals that in budding yeast cell volume itself rather than cell curvature seems to be the relevant parameter setting Cdc42-GTP cluster size and septin ring diameter. Moreover, we found that deletion of *BNI1* leads to increased septin ring diameters without affecting the cell-volume dependence of Cdc42-GTP cluster area, revealing that Cdc42-GTP cluster size and septin ring diameter are not strictly coupled. Instead, it suggests that septin ring diameter is established through the complex feedback dynamics of septin recruitment to the initial Cdc42-GTP cluster^11^, which is in line with the fact that manipulation of the polarization dynamics affects septin ring diameter^30^. Importantly, while we find that the septin ring diameter increases with cell volume throughout its presence (Supplementary Fig. 2a), the fact that mutations in *CDC42* can selectively affect septin ring diameter at the early stages of septin assembly^40^ suggests that studying the detailed evolution of ring size during the cell cycle will give important mechanistic insights.

While Okada et al. proposed a quantitative model linking septin ring diameter to Cdc42-GTP cluster size^11^, cell size has not been considered as a key determinant so far. However, since cell size is at least an implicit parameter in every model for cell polarization, the fact that septin ring diameter increases with cell volume provides a clear prediction that can be used as an independent test for already existing models aiming to explain septin ring formation.

More generally, our findings demonstrate that the budding yeast septin and contractile rings behave like other organelles whose size increases with cell size, including the nucleus^41,42^, mitochondria^43^, vacuoles^44^, and the mitotic spindle^45^. As for these other examples, this raises not only the question of how the ring diameter is mechanistically linked to cell volume, but also what determines optimal ring size. A priori, it is not obvious why ring size is regulated at all. Thus, the fact that in a constant environment cell size control constrains cell variability to a narrow range^46^ makes it conceivable that the relatively weak increase of ring diameter within the physiological cell size range is simply a by-product of a potential general tendency of macroscopic self-assembly processes to result in structures that scale with cell size. However, one interesting observation is the fact that bigger cells not only build bigger bud necks and grow larger buds, but also have bigger nuclei^41^. Since budding yeast undergoes closed mitosis, and septins have been shown to play a crucial role in building a nuclear diffusion barrier involved in asymmetric division^47^, one might speculate that a cell-volume-dependent septin ring diameter may be important for accurate segregation of the nucleus into the bud. Indeed, in mutant cells that repeatedly bud from the same site, decreased replicative lifespan and nuclear segregation defects have been suggested to result from the fact that the bud neck becomes narrower with each budding event^48^. Coordinating nuclear and septin ring diameter in cells of different ploidy may be particularly important considering that changes in ploidy go along with a roughly proportional change in cell volume. Linking both nuclear and septin ring diameter to cell volume may be a robust way for cells to indirectly coordinate these two size control processes.

Interestingly, deletion of *BNI1* alters the dependence of septin ring diameter both on cell volume and Cdc42-cluster size. The finding that Bni1—probably through its actin-regulating function—contributes to septin ring diameter without affecting Cdc42-GTP cluster size is a key prediction for future models. Moreover, it provides an opportunity to assess the impact of accurate ring size control on cell function and a first handle on revealing the molecular mechanism underlying septin ring size control. Establishing quantitative models that account for the cell-volume-dependent increase of the ring diameter and its dependence on Bni1 will potentially guide us to further genetic mutations that break the cell-volume dependence. We envision that such fruitful feedback between theory and experiment will make budding yeast ring diameter control a powerful and easily accessible model for organelle size control. Future studies may then enable us to understand with quantitative rigor the self-assembly process from the initial symmetry break to the establishment of a well-defined stable structure.

## Methods

### Yeast

*Strain construction:* All strains used in this study are congenic with W303 and constructed with standard methods. A strain list, as well as a list of primers used to construct and verify the strains, is available in the Supplementary information. β-estradiol-inducible strains are based on the system described by Ottoz et al.^27^. Strains with inducible Whi5 are based on the constructs established in^23^. The strain carrying inducible Cln1 was based on a strain constructed by Jennifer Ewald, which was obtained similar to strain JE611c in Ewald et al.^29^. To visualize Cdc42-GTP, we used the GIC2PBD(W23A)−1.5tdTomato-v2 construct described by Okada et al^11^.

*Growth conditions:* Prior to experiments, cells were cultured in synthetic complete liquid medium at 30 °C at low density to ensure exponential growth. As carbon source, we used either 2% glycerol 1% ethanol (SCGE, Figs. 1, 2, 3e–g, 5, 7–9; Supplementary Figs. 1, 2, 4) or 2% glucose (SCD, Figs. 3a–d, 4, 6, 8; Supplementary Fig. 3). Strains carrying β-estradiol-inducible *WHI5* were grown in the presence of the respective β-estradiol concentration for ~24 h before the start of the experiment to ensure steady state. Medium without β-estradiol was used during the live-cell microscopy experiments.

For the experiment described in Fig. 3b–d, the strain carrying β-estradiol-inducible *CLN1* was precultured in SCD medium containing 60 nM β-estradiol. Using 60 nM β-estradiol in the microfluidics setup (Cellasic) during the microscopy experiment turned out to be insufficient for maintaining cycling cells. Instead, a higher concentration of 500 nM β-estradiol was used. To release cells from the G1 arrest caused by β-estradiol removal, cells were first exposed to medium containing 5 μM β-estradiol for 15 min before reverting to the medium containing 500 nM β-estradiol.

### Live-cell microscopy

*Nikon Eclipse setup with custom microfluidics setup* *(data shown in Figs.* *1, 2, **3**a, e–g,* *4**–**9**; Supplementary Figs.* *1**–**4**)*: Live-cell time-lapse microscopy was performed in a custom made microfluidic device made of polydimethylsiloxane and a glass cover slip that allows trapping of cells in a dedicated region of interest, limiting colony growth in the *XY*-plane. Constant medium flow at 40 µl/min was applied, enabling imaging of colony growth over several generations. For aging experiments shown in Fig. 4, a different device, described by Goulev et al.^32^, was used, which allows imaging of mother cells trapped in a cavity for many generations (10–20 µl/min flow rate). A Nikon Eclipse Ti-E with NIS-Elements software, SPECTRA X light engine illumination and an Andor iXon Ultra 888 camera was used for epifluorescence microscopy. A plan-apo *λ* 100×/1.45 Na Ph3 oil immersion objective was used to take phase contrast and fluorescence images with a 3-min frame rate. For the experiments shown in Supplementary Fig. 1e, a plan-apo *λ* 60×/1.4Na Ph3 objective was used instead. For automated focusing, the built-in Nikon perfect focus system was used during the experiment. mCitrine fluorescence was imaged by exposure for 400 ms, illuminating with the SPECTRA X light engine at 504 nm and about 12 mW (20%) power. tdTomato and mKate2 fluorescence were imaged by exposure for 200 ms, illuminating with the SPECTRA X light engine at 556 nm and about 26 mW (10%) power. Identical settings were used for each of the experiments. Temperature control was achieved by setting both a custom made heatable insertion and an objective heater to 30 °C.

For control experiments shown in Supplementary Fig. 1b, cells were loaded in a custom made microfluidic device. For each position, we took epifluorescence images with settings identical to the time-lapse experiments, except that we varied the LED intensity from 3 to 24 mW (5–40%) power.

*Zeiss LSM800 setup with Cellasic microfluidics setup (data shown in Fig.* *3**b–d) and custom microfluidics setup (data shown in* *Supplementary Fig.* *1**a, c, d*): Live-cell time-lapse microscopy was performed in a Cellasic microfluidic device with Y04C plates. A Zeiss LSM800 microscope with Zen 2.3 (blue edition) software, Colibri 7 illumination and an Axiocam 506 mono camera was used for epifluorescence microscopy. A plan-apo 40×/1.3Na Ph3 oil immersion objective was used to take phase contrast and fluorescence images with a 3-min frame rate. Focusing was achieved using Definite Focus 2. mCitrine fluorescence was imaged by exposure for 10 ms with the Colibri 7 511 nm LED at 25% power. Temperature control was achieved by setting both an XL S1 heating unit and an objective heater to 30 °C.

For the control experiments shown in Supplementary Fig. 1a, c, d, cells were loaded in a custom made microfluidic device. For each position, we first took an epifluorescence image with settings identical to the time-lapse experiments. Within 1 min, we then switched to the confocal mode of the LSM800. For the experiments shown in Supplementary Fig. 1a, we took a 10 μm z-stack with 1 μm image separation of the same position, using the 488 nm laser at 0.15% power. For the experiments shown in Supplementary Fig. 1c, d, we acquired 12 μm z-stacks with 0.3 μm image separation, using the 589 nm laser at 0.3% power.

### Image analysis

*Cell segmentation:* For experiments shown in Figs. 1–8, Supplementary Figs. 1e and 2, cell segmentation based on phase contrast images was performed as described in detail by Doncic et al.^20^. The resulting cell segmentation was manually checked for each cell included in the analysis.

For aging experiments shown in Fig. 4, mother and daughter cells were manually segmented at the time point of cytokinesis as determined by visual inspection of the phase contrast time-lapse using the Matlab based Phylocell software developed in the Gilles Charvin lab^32^.

For experiments shown in Fig. 9 and Supplementary Fig. 4, cells were automatically segmented based on phase contrast images using Phylocell. Segmentation results were visually inspected and manually corrected if necessary.

*Calculation of cell volume and length along the major axis*: To calculate cell volume based on 2D phase contrast images, we first aligned the segmented cell area along its major axis. Next, we divided the cell into slices perpendicular to the major axis, each one pixel in width. To approximate cell volume, we then assumed rotational symmetry of each slice around its middle axis parallel to the cell’s major axis. We then calculated for each slice the volume of the resulting cylinder with one pixel in height and diameter the width of the respective slice. Finally, we summed the volumes of each cylinder to obtain total cell volume.

Length along the major axis was obtained using the Matlab function regionprops.

*Comparison between volume estimation using revolution and 3D reconstruction (Supplementary Fig.* *1c, d**)*. To validate the volume calculation method from 2D phase contrast images we compared it with an alternative method that relies on 3D reconstruction from z-stacks acquired with confocal microscopy. First, we performed image segmentation of the widefield phase contrast images to determine the revolution volume of each cell. Next, for each confocal image of each cell, we segmented the area delimited by the fluorescent intensity of mKate2 expressed from an *ACT1* promoter. Segmentation of each slice was achieved by hysteresis thresholding using the Python image processing library scikit-image. With this method, the pixels with intensity above a low threshold value are considered foreground only if they are connected to pixels with intensity above a high threshold value. This method ensures that bright pixels outside of the cell are not segmented. To avoid bias introduced by manual selection of low and high threshold values required for hysteresis thresholding, we determined the threshold values with automatic thresholding methods. In particular, the low threshold was calculated using the Li thresholding method^49^, while the high threshold was set to 90% of the maximum intensity value within the phase contrast segmented area. Finally, the total number of pixels of each slice was added to obtain the total number of voxels in the cell volume and converted to femtoliters.

3D visualization of the two methods (Supplementary Fig. 1d) was obtained using the Python library matplotlib.

*Comparison of cell volume and protein amount (Supplementary Fig.* *1e**)*: To test whether cell volume as obtained from phase contrast images correlates with total protein amount, we used fluorescence intensity of mKate2 expressed from an *ACT1* promoter as a proxy for cellular protein content. For each cell, we determined median mother cell volume during the time in which a septin ring is detected based on Cdc10-mCitrine fluorescence, as well as median total cellular mKate2 fluorescence during the same time. Background fluorescence was determined for each microscopy image as previously described^22^. Briefly, we applied a four-pixel average filter and the background was then taken to be the median pixel value of the non-cell area. Cellular autofluorescence in the mKate2 channel was estimated from still images of control cells without the *mKate2* allele, and found to be on the order of 1% of the mKate2 signal and therefore neglected.

*Analysis of the ring diameter (Figs.* *1**–**8**)*: For experiments shown in Figs. 1–8 and Supplementary Fig. 2, ring segmentation was performed in Fiji to automatically determine the position and orientation of the ring and obtain an intensity line profile along its major axis that was then further analyzed using Matlab. First, a threshold intensity was manually determined for each experiment to create a binary image from the fluorescence image. Next, for each cell to be analyzed, the first time point where the ring was present was manually determined, and a region of interest was selected that included no other ring during the period in which the ring of interest was present. The Fiji function Analyze Particles was then used to fit an ellipse to the ring, using a minimal ring area of ten pixels. The ring was analyzed for each following time point as long as it was identified by Analyze Particles. Cells for which the ring was identified for <5 frames, for which the ring was not in focus, or for which obvious segmentation errors occurred were rejected from the analysis. The ellipse fit was then used to obtain a brightness profile from the original fluorescence image along the major axis of the fitted ellipse using the Fiji function getProfile along a line 30 pixels left and right from the ellipse center (60 pixels in the case of inducible-Whi5 *bem2Δ* cells, and *och1Δ* cells grown on SCD), averaging over a width of ten pixels perpendicular to the major axis.

After applying a gliding average of three pixels to the brightness profile, and subsequent subtraction of the background defined as the mean of the three pixels most distant to the center at each side, the ring diameter was determined by measuring the distance between the points where the brightness profile rises the first time above half of the maximum brightness.

*Analysis shown in* *Fig.* *9* *and Supplementary Fig.* *4*

Analysis of the Cdc42 cluster: All parameters of the Cdc42-GTP cluster were calculated for the three frames centered around the frame with the peak Cdc42-GTP localization before bud emergence, which was determined by visual inspection.

To measure the Cdc42-GTP cluster area, a threshold approach similar to that used by Okada et al.^11^ was implemented. First, for each cell and each time point, a variable threshold was defined as the median value + 2 standard deviations for the fluorescence pixel intensities within the contour of the selected cell. Then, all pixels with a value higher than the threshold were counted as part of the cluster, and the cluster area was defined as the median number of pixels in the cluster during the three frames analyzed. Major and minor axes of the Cdc42 cluster were determined using the Matlab function regionprops for the largest object that is part of the cluster.

As an alternative approach similar to the one applied by Bonazzi et al. to measure the size of the Cdc42-GTP cluster in fission yeast^18^, we measured the length of the Cdc42-GTP cluster based on a fluorescence profile along the cell segmentation contour, consisting of 65 vertices. To account for the fact that the Cdc42-GTP cluster appears to be shifted toward the inside of the cell from the segmentation contour, we first applied a 5 × 5 maximum filter on the fluorescence image. A mean fluorescence profile was calculated based on the three selected frames. The length of the Cdc42-GTP cluster was defined as the width of the peak in the mean fluorescence profile above a threshold, which was defined as the median value of the individual thresholds of the three frames as described above. We visually inspected all length profiles and rejected cells from further analysis when our approach resulted in obvious artifacts, as, for example, caused by additional fluorescence signal from a Cdc42-GTP cluster in a neighboring cell.

Importantly, we find that both approaches described above yield highly correlated results, which confirm that Cdc42-GTP cluster diameter increases with cell volume. Specifically, we find that the lengths of the major and minor axes of the Cdc42-GTP cluster area increase with cell volume (Supplementary Fig. 4a–d), which highlights that the increase of the cluster area reflects an increase of the cluster diameter. Moreover, we observe a strong correlation (*R* = 0.58 for wild-type cells, *R* = 0.79 for *bni1Δ* cells) between the Cdc42-GTP cluster length measured along the cell contour and the length of the major axis of the Cdc42-GTP cluster area (Supplementary Fig. 4e, f). We therefore conclude that Cdc42-GTP cluster area is a good measure that can be used to identify changes in the Cdc42-GTP cluster diameter.

Analysis of the septin ring diameter and the local radius of curvature: The septin ring diameter based on Cdc10-mCitrine fluorescence as well as mother cell volume and the local radius of curvature were calculated from the five frames centered around the frame corresponding to clear bud emergence, which was defined by visual inspection.

As described for the Cdc42-GTP cluster length, a fluorescence profile along a cell segmentation contour was taken. Since the septin fluorescence signal tends to be well-aligned with the cell contour defined by the cell segmentation, we found that a 3 × 3 mean filter is sufficient for the measurement of septin ring diameter. After applying the 3 × 3 mean filter on the fluorescence image, a mean fluorescence profile was calculated based on the five selected frames. The septin ring diameter was then defined as the full width at half maximum, where we defined the minimum signal in the fluorescence profile as the baseline. We visually inspected all length profiles and rejected cells from further analysis when our approach resulted in obvious artifacts, as, for example, caused by additional fluorescence signal from a septin ring in a neighboring cell.

To measure the local radius of curvature, a circle was fitted to the seven vertices of the cell contour centered around the position of the maximum of the Cdc10-mCitrine fluorescence intensity profile. Then, the corresponding radius was calculated for each of the selected frames centered around bud emergence, and the median value was used for the analysis.

*Two-variable linear regressions*: Two-variable linear regressions with constant offset were performed using the Matlab function glmfit. For each parameter, linear regression provides a coefficient and a corresponding *p* value, which tests whether the coefficient is statistically significantly different from 0. If the coefficient is statistically significantly different from 0, it implies that the corresponding parameter adds predictive information to the model.

For the area of the Cdc42 cluster, *A*, we find that the cube root of cell volume, $V$, is the predictive parameter, while local curvature, $R_C$, adds no significant predictive information. Specifically, for wild-type cells we obtain $A = 0.47V^{1/3} + 0.006R_C/V^{1/3} - 0.53$, with the *p* values for the three coefficients being 2.3 · 10^−21^, 0.94, and 10^−4^, respectively. For *bni1Δ* cells, we obtain $A = 0.62V^{1/3} - 0.08R_C/V^{1/3} - 1.28$, with the *p* values for the three coefficients being 1.2 · 10^−20^, 0.43, and 5.4 · 10^−10^, respectively. Note that for both wild-type and *bni1Δ* cells we observed a strong correlation between $V^{1/3}$ and $R_C/V^{1/3}$ (*p* < 10^−18^ and *p* < 10^−17^, respectively).

For the septin ring diameter, *d*, we find that the cube root of cell volume is the main predictive parameter, with local curvature adding additional predictive information. Specifically, for wild-type cells we obtain $d = 0.27V^{1/3} - 0.17R_C/V^{1/3} + 0.74$, with the *p* values for the three coefficients being 6.8 · 10^−12^, 0.012, and 2.9 · 10^−8^, respectively. For *bni1Δ* cells, we obtain $d = 0.27V^{1/3} - 0.17R_C/V^{1/3} + 1$, with the *p* values for the three coefficients being 1.5 · 10^−9^, 0.037, and 3.9 · 10^−10^, respectively.

*Correlation analysis*: Correlation coefficients and corresponding *p* values where obtained using the Matlab function corrcoef.

### Statistics and reproducibility

All measurements except for control experiments shown in Fig. S1 are based on at least two independent biological replicates. For some slow growing mutants, more than two experiments were necessary to obtain a sufficient number of single-cell measurements. Results from individual replicates were compared, and no major differences were noted.

### Reporting summary

Further information on research design is available in the Nature Research Reporting Summary linked to this article.

## Supplementary information

Supplementary Information

Reporting Summary

## Data Availability

Yeast strains as well as microscopy raw files are available upon reasonable request. Source Data are provided with this paper.

## Source data

Source Data

## Code availability

Additional information on image analysis approaches described in “Methods” and previous publications is available upon reasonable request.
