# Supplementary material for: Cell size sets the diameter of the budding yeast contractile ring
Source: Nat Commun. 2020 Jun 11;11:2952. doi: 10.1038/s41467-020-16764-x (PMC7289848; doi:10.1038/s41467-020-16764-x)
Supplement: Supplementary file 1 — Supplementary Information [file 41467_2020_16764_MOESM1_ESM.pdf]

Supplementary information:

Supplementary figures

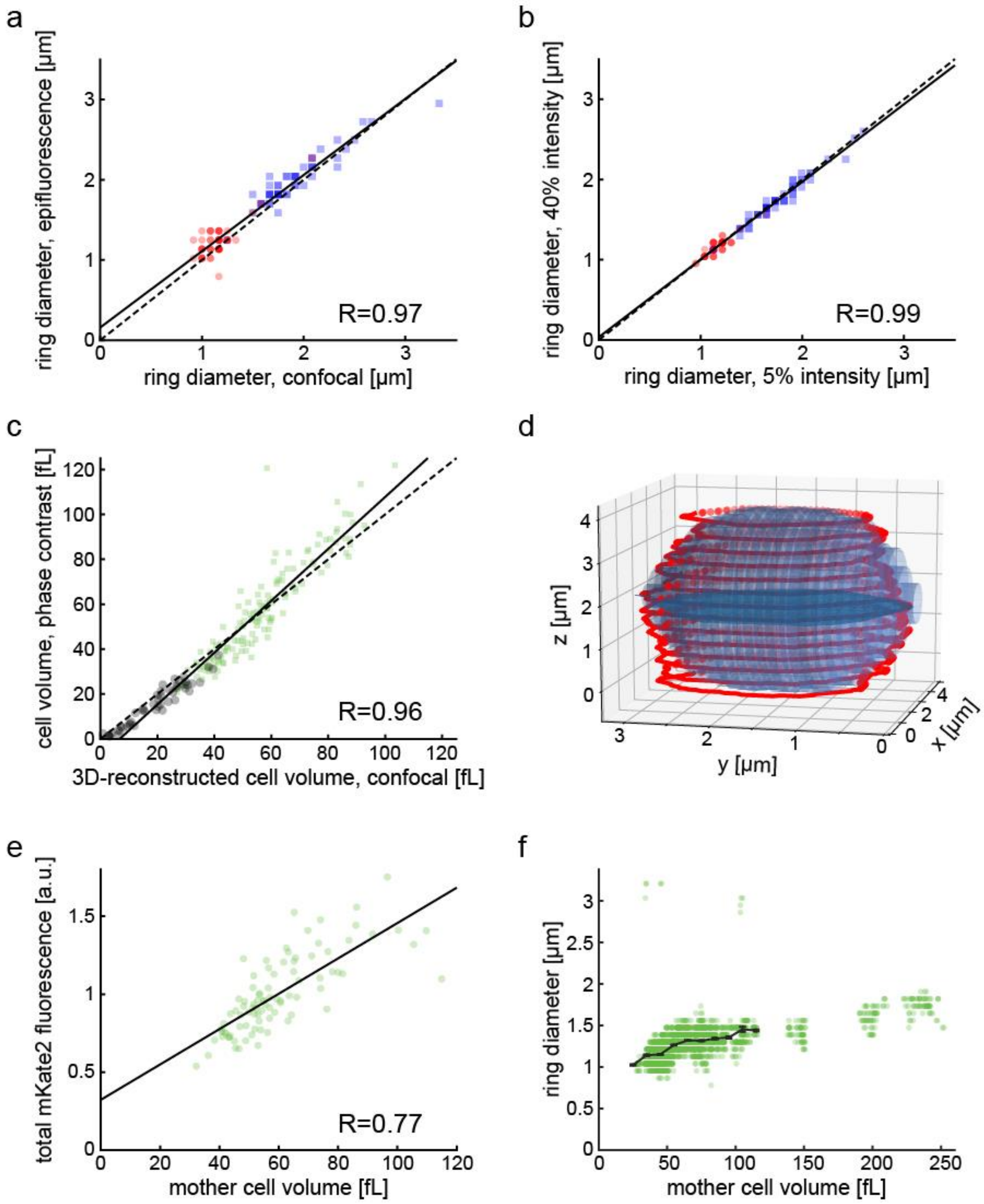

Supplementary Fig. 1: a) Whi5-inducible cells carrying Cdc10-mCitrine grown in the absence (red circles, 50 cells) and presence of 30 nM β-estradiol (blue squares, 50 cells) were imaged within one minute with epifluorescence (single image) and confocal microscopy (10 µm Z-stack, 1 µm

steps between focal planes) using a hybrid Zeiss LSM800 setup. Septin ring diameters were determined by manually drawing line profiles along septin rings in both epifluorescence images and maximum projections of the confocal Z-stacks. For each cell, the ring diameter as determined from epifluorescence microscopy is shown as a function of the ring diameter determined from confocal microscopy. **b)** Whi5-inducible cells carrying Cdc10-mCitrine grown in the absence (red circles, 50 cells) and presence of 30 nM β-estradiol (blue squares, 50 cells) were imaged with the Nikon Eclipse setup at lower (5%) and higher (40%) LED intensity compared to settings chosen for the experiments shown in the main text (20%). Septin ring diameters were determined by manually drawing line profiles along septin rings. For each cell, the ring diameter as determined from epifluorescence microscopy at high illumination intensity is shown as a function of the ring diameter determined at low intensity. **c)** Cell volume as determined from phase contrast images is shown as a function of cell volume determined from 3D-reconstruction based on confocal fluorescence microscopy for cells expressing mKate2 from an *ACT1* promoter. Buds (black circles, n=49) and G1 and mother cells not including the bud (green squares, n=138) were included in the analysis. **d)** Both 3D reconstructed volume (53.4 fL, red) and volume approximated from phase contrast (56 fL, blue) is shown for a representative cell. Data shown in a-d are based on single experiments. **e)** Median total mKate2 fluorescence in mother cells is shown as a function of median mother cell volume based on phase contrast during the time when the septin ring is detected in cells expressing mKate2 from an *ACT1* promoter (n=89 pooled from 3 independent experiments). To account for intensity variations across experiments, mKate2 fluorescence was normalized for each experiment on the average fluorescence at the mean cell volume. Solid lines in a-c) and e) show linear fits, dashed lines show identity lines for comparison. **f)** Raw data corresponding to Fig. 1e. While Fig 1e shows for each cell the median volume and ring diameter during the time when the ring is detected, raw data for all time points are shown here. Error bars denote means and standard errors. Cells were grown on SCGE. Source data are provided as a Source Data file.

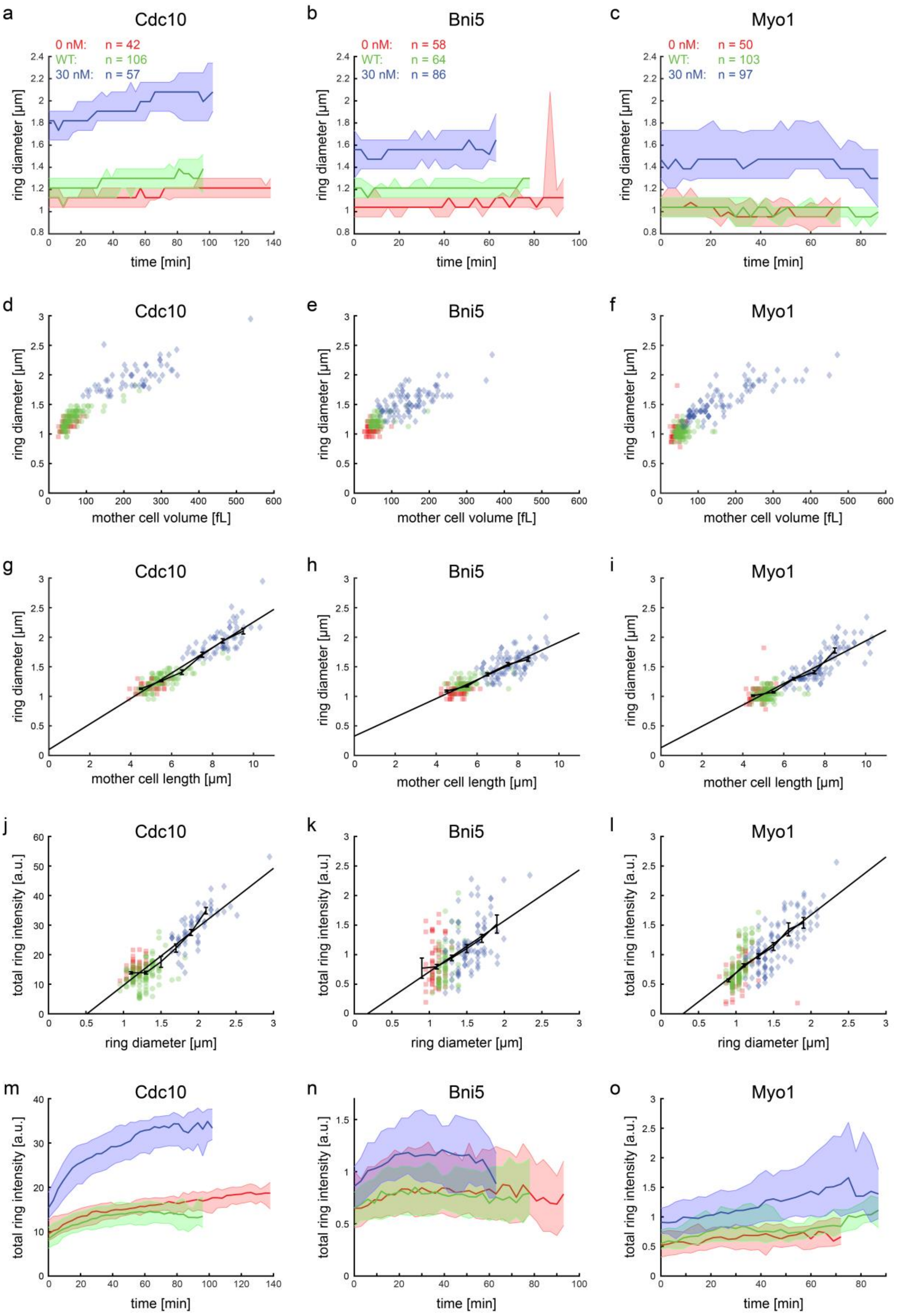

a
Cdc10
0 nM: n = 42
WT: n = 106
30 nM: n = 57
ring diameter [µm]
time [min]
b
Bni5
0 nM: n = 58
WT: n = 64
30 nM: n = 86
ring diameter [µm]
time [min]
c
Myo1
0 nM: n = 50
WT: n = 103
30 nM: n = 97
ring diameter [µm]
time [min]
d
Cdc10
ring diameter [µm]
mother cell volume [fL]
e
Bni5
ring diameter [µm]
mother cell volume [fL]
f
Myo1
ring diameter [µm]
mother cell volume [fL]
g
Cdc10
ring diameter [µm]
mother cell length [µm]
h
Bni5
ring diameter [µm]
mother cell length [µm]
i
Myo1
ring diameter [µm]
mother cell length [µm]
j
Cdc10
total ring intensity [a.u.]
ring diameter [µm]
k
Bni5
total ring intensity [a.u.]
ring diameter [µm]
l
Myo1
total ring intensity [a.u.]
ring diameter [µm]
m
Cdc10
total ring intensity [a.u.]
time [min]
n
Bni5
total ring intensity [a.u.]
time [min]
o
Myo1
total ring intensity [a.u.]
time [min]

Supplementary Fig. 2: a-c) The median ring diameter (thick lines) as quantified from mCitrine-tagged Cdc10 (a), Bni5 (b), and Myo1 (c) is shown as a function of the time since the first frame where the ring was detected for analysis. Error bars show 25 and 75 percentiles. Data are shown for the time ranges during which at least 15 cells of the respective condition were included in the analysis. In the case of Myo1, we typically observe ring contraction at the end of the cell cycle. However, due to fast contraction dynamics and the rather low time resolution (3 min frame rate), this usually only affects the last timepoints analyzed and has therefore no strong effect on the calculation of the median ring diameter during Myo1 presence. **d-f)** Linear plots corresponding to Fig. 2c,e,g showing ring diameter as a function of cell volume. **g-i)** Median ring diameter during the time when the ring is detected is shown as a function of the median cell length along the major axis. Data from different conditions are pooled and linear fits, as well as binned means with standard error, are shown for each tagged protein. **j-l)** Median total fluorescence intensity (integrated line profile intensity after background subtraction) during the time when the ring is detected is shown as a function of median ring diameter during that time (red squares: 0 nM β-estradiol; green circles: wild-type; blue diamonds: 30 nM β-estradiol). Data from different conditions are pooled and binned means with standard error are shown for each tagged protein. **m-o)** Median total fluorescence (thick lines) is shown as a function of the time since the first frame where the ring was detected for analysis. Error bars show 25 and 75 percentiles. Data are shown for the time ranges during which at least 15 cells of the respective condition were included in the analysis. Solid lines in d)-l) show linear fits to the pooled data. Cells were grown on SCGE. Source data are provided as a Source Data file.

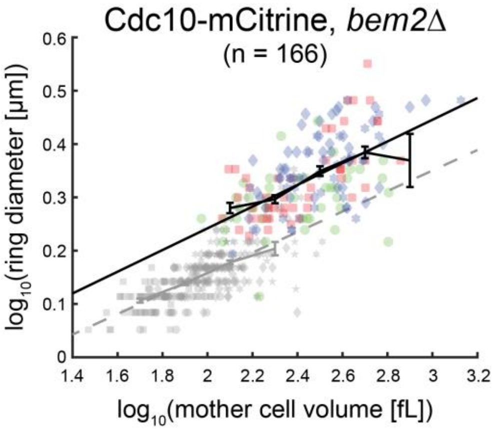

Supplementary Fig. 3: Septin ring diameter based on Cdc10-mCitrine fluorescence is shown as a function of cell volume for *bem2Δ* cells and compared to data obtained for wild-type cells in Fig 3a (grey). Cell volume was controlled through β-estradiol-dependent expression of Whi5 (red squares: 0 nM β-estradiol; green circles: non-inducible-Whi5 strain; blue: 60 nM (diamonds), 120 nM β-estradiol (hexagrams)). For each cell, the median ring diameter during the time when the ring is detected is shown as a function of the median cell volume during that time. Data from different conditions are pooled (166 cells pooled from 4 independent experiments) and a linear fit to the double-logarithmic data as well as binned means with standard error are shown. Source data are provided as a Source Data file.

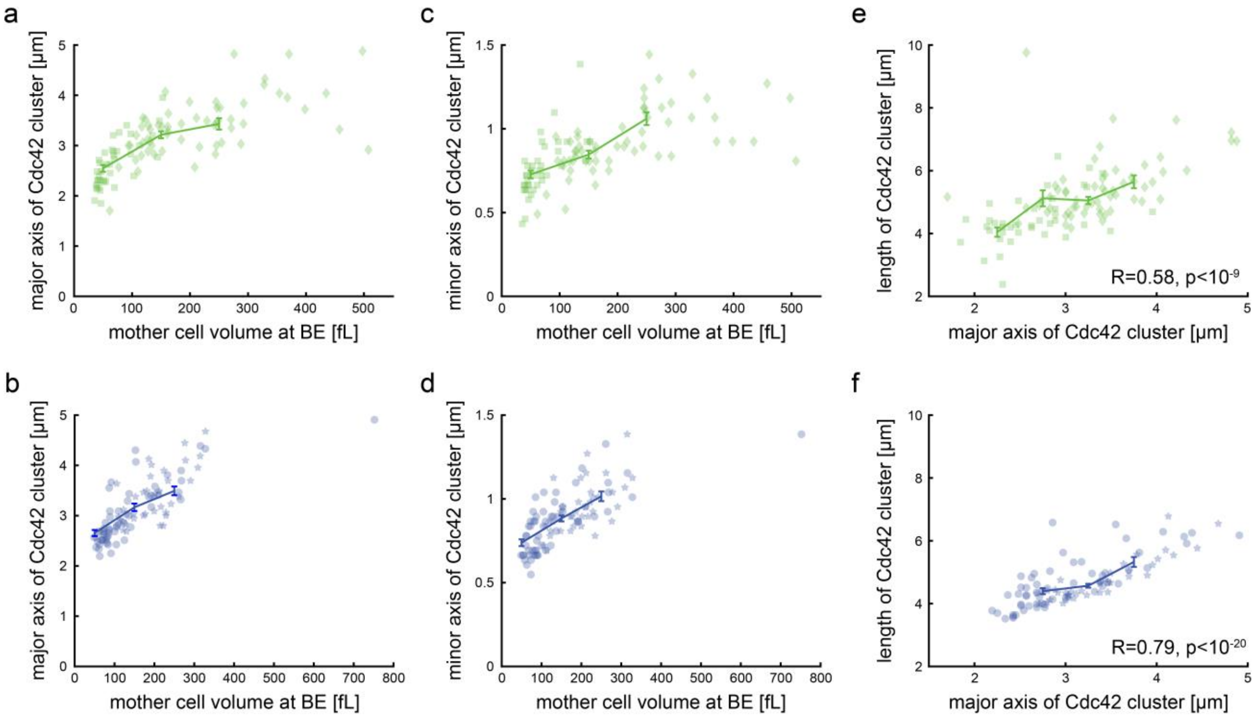

Supplementary Fig. 4: a-d) The lengths of the major (a-b) and minor (c-d) axes of the Cdc42-GTP cluster at peak intensity in wild-type (a&c; squares: 0 nM β-estradiol, diamonds: 30 nM β-estradiol) and *bni1Δ* (b&d; circles: 0 nM β-estradiol, pentagrams: 30 nM β-estradiol) cells carrying inducible Whi5 shown in Fig. 9c increase with mother cell volume at bud emergence. **e-f)** The length of the Cdc42-GTP cluster measured from the fluorescence intensity line profile along the cell contour is correlated with the length of the major axis of the Cdc42-GTP cluster for wild-type (e) and *bni1Δ* (f) cells. Data from different conditions are pooled and binned means with standard error are shown for each tagged protein. Cells were grown on SCGE. Source data are provided as a Source Data file.

Strain list

Name	Genotype	Origin	Figures
KSY195-1	*Mat α; ADE2 cdc10::CDC10-mCitrine-ADH1term-HIS3*	This study	2, 3-6, 8, S1-3
KSY236-2	*Mat a; ADE2 cdc10::CDC10-mCitrine-ADH1term-TRP1*	This study	2, 5, 8, S2
KSY234-1	*Mat a; ADE2 his3::LexA-ER-AD-TF-HIS3 whi5::kanMX6-LexApr-WHI5-ADH1term-LEU2 cdc10::CDC10-mCitrine-ADH1term-TRP1*	This study	2, 3, 5, 6, 8, S1-3
KSY196-1	*Mat α; ADE2, bni5::BNI5-mCitrine-ADH1term-HIS3*	This study	2, S2
KSY203	*Mat α; ADE2 his3::LexA-ER-AD-TF-HIS3 whi5::kanMX6-LexApr-WHI5-mCherry-ADH1term -LEU2 bni5::BNI5-mCitrine-ADH1term-HIS3*	This study	2, S2
KSY121-1a	*Mat α; ADE2 myo1::MYO1-mCitrine-ADH1term-HIS3*	This study	2, 5, S2
KSY197-2	*Mat α; ADE2 myo1::MYO1-mCitrine-ADH1term-TRP1*	This study	2, 5, S2
KSY192-1	*Mat α; ADE2 his3::LexA-ER-AD-TF-HIS3 whi5::kanMX6-LexApr-WHI5-mCherry-ADH1term-LEU2 myo1::MYO1-mCitrine-ADH1term-HIS3*	This study	2, S2
KSY242-2	*Mat α; ADE2 TRP1 cln1Δ cln2Δ cln3::LEU2 LexApr-CLN1-LEU2 his3::LexA-ER-AD-HIS3 cdc10::CDC10-mCitrine-ADH1term-URA3*	This study	3
KSY287-9	*Mat α; ADE2 cdc10::CDC10-mCitrine-ADH1term-HIS3 cln3Δ::KlacURA3*	This study	3

KSY286-15	*Mat α; ADE2 cdc10::CDC10-mCitrine-ADH1term-HIS3 cln1Δ::KlacURA3*	This study	3
KSY288-3	*Mat α; ADE2 cdc10::CDC10-mCitrine-ADH1term-HIS3 clb5Δ::KlacURA3*	This study	3, 8
KSY237-1	*Mat a/α; ADE2/ADE2 cdc10::CDC10-mCitrine-ADH1term-HIS3/cdc10::CDC10-mCitrine-ADH1term-TRP1*	This study	5, 7
KSY199-1	*Mat a/α; ADE2/ADE2 myo1::MYO1-mCitrine- ADH1term-HIS3/myo1::MYO1-mCitrine-ADH1term-TRP1*	This study	5
KSY243-2	*Mat a/α; ADE2/ADE2 his3/his3::LexA-ER-AD-TF-HIS3 WHI5/whi5::kanMX6-LexApr-WHI5-ADH1term-LEU2 cdc10::CDC10-mCitrine-ADH1term-TRP1/cdc10:: CDC10-mCitr-ADH1term-URA3*	This study	5, 7
KSY258-1&2	*Mat α; ADE2 cdc10::CDC10-mCitrine-ADH1term-HIS3 rga1Δ::KlacURA3*	This study	6
KSY264-2	*Mat α; ADE2 cdc10::CDC10-mCitrine-ADH1term-HIS3 rga1Δ::KlacURA3 rga2Δ::CglaTRP1*	This study	6
KSY267-1&2	*Mat α; ADE2 cdc10::CDC10-mCitrine-ADH1term-HIS3 rga1Δ::KlacURA3 rga2Δ::CglaTRP1 bem3Δ::CglaLEU2*	This study	6
KSY238-1a	*Mat α; ADE2 bem2::natMX6 cdc10::CDC10-mCitrine-ADH1term-HIS3*	This study	6
KSY239-4	*Mat a/α; ADE2/ADE2 cdc10::CDC10-mCitrine-ADH1term-HIS3/cdc10::CDC10-mCitrine-ADH1term-TRP1 CDC42/cdc42::KlacURA3*	This study	7
KSY268-1&2	*Mat a/α; ADE2/ADE2 cdc10Δ::klacURA3/cdc10::CDC10-mCitrine-ADH1term-TRP1*	This study	7
KSY260-2	*Mat α; ADE2 cdc10::CDC10-mCitrine-ADH1term-HIS3 bni1Δ::KlacURA3*	This study	8
KSY266-1	*Mat α; ADE2 cdc10::CDC10-mCitrine-ADH1term-HIS3 arp8Δ::KlacURA3*	This study	8

KSY284-5&6	*Mat α; ADE2 cdc10::CDC10-mCitrine-ADH1term-HIS3 och1Δ::KlacURA3*	This study	8
KSY265-1	*Mat α; ADE2 cdc10::CDC10-mCitrine-ADH1term-HIS3 spt20Δ::KlacURA3*	This study	8
KSY235-4	*Mat a; ADE2 his3::LexA-ER-AD-TF-HIS3 whi5::kanMX6-LexApr-WHI5-ADH1term-LEU2 cdc10::CDC10-mCitrine-ADH1term-TRP1 ura3::YIp211-GIC2PBD(W23A)-1.5tdTomato-v2-URA3*	This study	9, S4
KSY269-5&6	*Mat a; ADE2 his3::LexA-ER-AD-TF-HIS3 whi5::kanMX6-LexApr-WHI5-ADH1term-LEU2 cdc10::CDC10-mCitrine-ADH1term-TRP1 ura3::YIp211-GIC2PBD(W23A)-1.5tdTomato-v2-URA3 bni1Δ::natMX6*	This study	9, S4
KSY282-2	*Mat a; ADE2 cdc10::CDC10-mCitrine-ADH1term-TRP1 HIS3::Act1pr-mKate2-ADH1term-HIS3*	This study	S1
KSY281-10	*Mat a; ADE2 bem2::natMX6 his3::LexA-ER-AD-TF-HIS3 whi5::kanMX6-LexApr-WHI5-ADH1term-LEU2 cdc10::CDC10-mCitrine-ADH1term-TRP1,*	This study	S3

Primer list

Name	Descriptive name	Sequence
KP145	SacI_ADH1term_R	accGAGCTCCCGGTAGAGGTGTGGTCAATAAGAGC
KP155	SacI-MyoI-5201_F	cgcGAGCTCCAAGGAATGGGGAGTTGGTTAAGACATTAC
KP156	BamHI_Myo1_R	cacGGATCCACTGAAAATTTTACTCTGTGCATTGTTACTATCAATATTT TTCG
KP180	mCitr_200_F_qPCR	CGGCCTGATGTGCTTCGCC
KP181	mCitr_400_R_qPCR	CCTCCTTGAAGTCGATGCCC
KP184	Myo1_4500_F	GAACATATTCATGCACTAAAACAAGCTGAAGAGG
KP254	SacI-Cdc10-481_F	cgcGAGCTCGACGTTGAAGCCTTGAAAAGATTGACAGAAATAG
KP255	BamHI_Cdc10_R	cacGGATCCACGTTGAATGGCGTTGCTAGACATATGAG
KP258	SacI-Bni5-755_F	cgcGAGCTCGTTTAGCTGATCAAACACCCCATGACG
KP259	BamHI_Bni5_R	cacGGATCCTTTAGTTCCAATCCAAAATTGGCCATTTTCGCC
KP265	Cdc10_F	ATGGATCCTCTCAGCTCAGTACAGCC
KP266	Bni5_F	ATGGGCTTGGACCAGGACAAGATAAAGAAG
KP364	Cdc42-80bphom-F1	CCTGAGGAGATAGGTTAACAAACGAATTAGAGAAGCAAAACTTATA AAACAAGAAATAAACGTATTAGCTCTTCCACAAAcggatccccgggttaatt aa
KP365	Cdc42-80bphom-R1	GATAATAAAAGGATAGGAAGGTGTATATATAAGTTAATTTTAGATAT AGATTAAGAAAAGATGGGCATATACTAATATGAgaattcgagctcgtttaa ac
KP366	Cdc42seq_F	GCAATAGGTTTCCTTTGTCG
KP367	Cdc42seq_R	GAGAATCAACTCTGAGCAAAGC
KP368	Bem2-66bphom-F	GAACACACGGTCGTCGTGCCTTTTCTGGATAGACACAAAAAAAACAA ATAACGAAGCAGGAGTCTA GTTTAGCTTGCCTTGTCCC
KP369	Bem2-75bphom-R	CTCAAACCCTCGATAGGCGGGACCAAATTTTTTCTCTCTCAGCAGTGG ATTGTATACATTTACCACGAAAATTGT ACTGGATGGCGGCG
KP370	Bem2seq_F	CGTTCCCGCTATACGC
KP371	Bem2seq_R	GGAGAAGAGGCAGAATTCC
KP429	Rga1-74bp-F1	CTCTATTATAGCTTTTTGTACAAGACAAGGATAGCTGATTCAGGTACT AGTGGTGGAGAGAGCGGCATATTAAAcggatccccgggttaattaa
KP430	Rga1-80bp-R1	CCTGCTTAAGTCTGCGATTAAAAAAATAACGTTTCGATACAGTTCATA TAAGGCGGCTCAATGCAGAACCGAGGATAGCGgaattcgagctcgtttaaa c
KP434	Bni1_71bp_F1	CACGCCACATAAAGAAGGCACCTGAACCTTTTCAACAAACGAGAAGC AAGAAAGGAAGAGAAGGAAAGGAAcggatccccgggttaattaa
KP435	Bni1_80bp_R1	CGTGACTATTTCTTTGTACTAGTGCTTGTTTGGATGTTTGTTTTGGTAT TACTGTTGTCATAATTTTTTGGTTTAATATTgaattcgagctcgtttaaac
KP442	Rga1_seq_F	GATCAGTCAACATTCTTGATCA
KP443	Rga1_seq_R	CAACGCTTACAGGTGTCC
KP446	Bni1_seq_F	CGTCTCATACAGTTGGGC
KP447	Bni1_seq_R	CACATGGAAACAATGGCAC
KP454	Spt20_80bp_F1	GATAAAAGAATGAGAAAAGTAGCCTTCAAGAGGATTAGGAAGGAAT AGTTACGGTTAATTTGCGCCTATATATTTCAGGGcggatccccgggttaatt aa

KP455	Spt20_Cterm64bphom_R1	TTATGACATTGTAGTAGAAGAGGGCGTGCTACTTGCGCTCTGTTTCTTCTTAGTCATTCTTTTCgaattcgagctcgtttaaac
KP459	Rga2-78bp-F1	CCAAGAGTTCATTGTACTTTTAATAAAGTGAAATATAACGTAGCATCTCAAGAGCAAGGAGATTTTGATGAAAAAAATcggatccccgggttaattaa
KP460	Rga2-80bp-R1	CTATTTTCTTACTTTATTCTTTTTTCATATGATTTCTTATTTAATCTATCCTATGTTTATTTAACTTTTGCAAATCTGTAgaattcgagctcgtttaaac
KP461	Bem3-80bp-F1	CACGTTTGTTGAAACACTGGCTGCTAGGAGTATCTGTGTGTATATTCTAGAATAAACTCACACTCAACTAACAGCACGCAcggatccccgggttaattaa
KP462	Bem3-80bp-R1	CTCTATACATCTCGCCCTCTTTCTATCATTAAATCAATGGAGGTTTACTGGCAACGTTATATTTCTACAATTTTAGACCAgaattcgagctcgtttaaac
KP463	Rga2_seq_F	GAAAATTGGCATTCCTTGGAG
KP464	Rga2_seq_R	CCAAGAAAAGACTTCACCAC
KP465	Spt20_seq_F	CCGCACCATTAATAACTAACTAAC
KP466	Spt20_seq_R	GTGAGATGCCCAAGTGAG
KP467	Arp8_seq_F	GCAGCAACTTTGGGTCC
KP468	Arp8_seq_R	GGACGCCTTCAAGTTGTGC
KP469	Cdc10_80bp_F1	TACTTAACTTTTTTCAGGCAAAGACAAGAAAATACAAGGCCAAGCCCCACGGTTACTACAAGCACTCTATAAATATATTAcggatccccgggttaattaa
KP470	Cdc10_80bp_R1	GTTACGGTGTTTTCATATAGAATAATTTGAGAATTCTTAATAACATAAGATATATAATCACCACCATTCTTATGAGATgaattcgagctcgtttaaac
KP471	Cdc10_seq_F	GCTTTCTGAATGCTTGCG
KP472	Cdc10_seq_R	CAAACGAGAAGGTGATAGCTG
KP473	Bem3_seq_F	CCGTTTACCTTTGTGTGTTAG
KP474	Bem3_seq_R	CGACCATTTGGTCAAGAAG
KP475	Bni1_71bphom-F	CACGCCACATAAAGAAGGCACCTGAACCTTTTCAACAAACGAGAAGCAAGAAAGGAAGAGAAGGAAAGGAA GTTTAGCTTGCCTTGTCCC
KP476	Bni1_80bphom-R	CGTGACTATTTCTTTGTACTAGTGCTTGTTTGGATGTTTGTTTTGGTATTACTGTTGTCATAATTTTTTGGTTTAATATT ACTGGATGGCGGCG
KP522	Och1_78bp_F1	CTTTGATTCCGTTTTCATTTCAAGAGCAATAATAGCAATTTGGAAAAAGAAAGCAAGTAAAAGAAAGAAGAGATCcggatccccgggttaattaa
KP523	Och1_80bp_R1	CATGATTAAGGATATGAAGAAGAAAGGAATAACTAGGAATAAATTTTATTTAGAGAGGGTATGATGAAAGGAGAGCCTCGgaattcgagctcgtttaaac
KP524	Och1_seq_F	GGTAGCTTGGTAGCCAC
KP525	Och1_seq_R	GCAGTGTTACAGAAGCACTG
KP538	Cln3_72bp_F1	GCATTTCTTACATTCCATTGCATCTCCCTTTTACTCTCGTTCAAGACACTGATTTGATACGCTTTCTGTACGcggatccccgggttaattaa
KP539	Cln3_78bp_R1	GATCATTAATGTATGTTAACGTATTTGCTTTGCAAATTTTAATTTATTTGTTGTTAAATGCATTTTTTTTTTGTCGTTgaattcgagctcgtttaaac
KP540	Cln3_seq_F	CAAGCATCCATCCGAGTC
KP541	Cln3_seq_R	GTAATCGTATTTTAGGTTGTGC
KP542	Cln1_80bp_F1	CAATAATAGCAATTAAATAAAATAGCACTACCACCACTCCACTGCTCGTTAGCTATTTCTGTAAAATAAATAAAAAGATCcggatccccgggttaattaa
KP543	Cln1_77bp_R1	GTCATTATTACTTACGATGGAAAAGCGTAGTATTCCGTTATTAATTAAGTATATATGTAGGCTTGATGAGAAAATGGgaattcgagctcgtttaaac
KP544	Cln1_seq_F	CCAAGGAGTTCTTCGTTCG
KP545	Cln1_seq_R	GGTATATTACTATTCAGCTGGTTTC

KP550	Clb5_80bp_F1	CACAAAGCAAAATAAGCTTAATAATTAGCAGTAACGCGCTTTTCCCTG TATTTAAAGCCGCTGAACACCTTTACTGAACAcggatccccgggttaattaa
KP551	Clb5_70bp_R1	CCTTTTAGTTCAGCAAAAAGAAAAGAAAATGTAAAGAGTATGCGAAT TCATGAGCATTACTAGTACTAATgaattcgagctcgtttaaac
KP552	Clb5_seq_F	CTTGGAACTAATTCTTAAGCTTCTC
KP553	Clb5_seq_R	GATGATAATAGTAGTAATACTGGTGG
