# Supplementary material for: Cell size sets the diameter of the budding yeast contractile ring
Source: Nat Commun. 2020 Jun 11;11:2952. doi: 10.1038/s41467-020-16764-x (PMC7289848; doi:10.1038/s41467-020-16764-x)
Supplement: Supplementary file 2 — Reporting Summary [file 41467_2020_16764_MOESM2_ESM.pdf]

nature research

Corresponding author(s): Kurt Schmoller

Last updated by author(s): May 4, 2020

Reporting Summary

Nature Research wishes to improve the reproducibility of the work that we publish. This form provides structure for consistency and transparency in reporting. For further information on Nature Research policies, see Authors & Referees and the Editorial Policy Checklist .

Statistics

For all statistical analyses, confirm that the following items are present in the figure legend, table legend, main text, or Methods section.

n/a	Confirmed	
☐	☒	The exact sample size (*n*) for each experimental group/condition, given as a discrete number and unit of measurement
☒	☐	A statement on whether measurements were taken from distinct samples or whether the same sample was measured repeatedly
☐	☒	The statistical test(s) used AND whether they are one- or two-sided<br>*Only common tests should be described solely by name; describe more complex techniques in the Methods section.*
☐	☒	A description of all covariates tested
☐	☒	A description of any assumptions or corrections, such as tests of normality and adjustment for multiple comparisons
☐	☒	A full description of the statistical parameters including central tendency (e.g. means) or other basic estimates (e.g. regression coefficient) AND variation (e.g. standard deviation) or associated estimates of uncertainty (e.g. confidence intervals)
☐	☒	For null hypothesis testing, the test statistic (e.g. *F*, *t*, *r*) with confidence intervals, effect sizes, degrees of freedom and *P* value noted<br>*Give P values as exact values whenever suitable.*
☒	☐	For Bayesian analysis, information on the choice of priors and Markov chain Monte Carlo settings
☒	☐	For hierarchical and complex designs, identification of the appropriate level for tests and full reporting of outcomes
☐	☒	Estimates of effect sizes (e.g. Cohen's *d*, Pearson's *r*), indicating how they were calculated

Our web collection on statistics for biologists contains articles on many of the points above.

Software and code

Policy information about availability of computer code

Data collection	Zeiss (Zen 2.3 (blue edition)) and Nikon (NIS-Elements 4.51.01 (Build) and 5.02.01 (Build 1270)) microscope software
Data analysis	Fiji (ImageJ 1.52e), Matlab R2016a & R2018a, Phylocell (version from 7 April 2017 https://github.com/gcharvin/phyloCell)

For manuscripts utilizing custom algorithms or software that are central to the research but not yet described in published literature, software must be made available to editors/reviewers. We strongly encourage code deposition in a community repository (e.g. GitHub). See the Nature Research guidelines for submitting code & software for further information.

Data

Policy information about availability of data

All manuscripts must include a data availability statement. This statement should provide the following information, where applicable:

- Accession codes, unique identifiers, or web links for publicly available datasets
- A list of figures that have associated raw data
- A description of any restrictions on data availability

Yeast strains as well as microscopy raw files are available upon reasonable request.

Field-specific reporting

Please select the one below that is the best fit for your research. If you are not sure, read the appropriate sections before making your selection.

☒ Life sciences ☐ Behavioural & social sciences ☐ Ecological, evolutionary & environmental sciences

For a reference copy of the document with all sections, see nature.com/documents/nr-reporting-summary-flat.pdf

Life sciences study design

All studies must disclose on these points even when the disclosure is negative.

Sample size	All measurements except for control experiments shown in Fig. S1 are based on at least two independent biological replicates. No sample size calculation was performed. Instead, we aimed to analyze at least 30 cells for each strain, which provides enough single cell measurements to robustly detect major phenotypes. For some slow growing mutants more than two experiments were necessary to obtain a sufficient number of single cell measurements.
Data exclusions	No experiments were excluded from analysis.
Replication	All measurements except for control experiments shown in Fig. S1 are based on at least two independent biological replicates. Before pooling data, replicate experiments were always compared and no pronounced differences were noted. Fig. S1, which controls for technical rather than biological questions, is based on a single biological replicate.
Randomization	Strains and conditions that were directly compared were typically measured in parallel in a single microfluidic device, making further randomization not applicable.
Blinding	Due to the obvious phenotypes of Whi5-induced cells and mutant strains, blinding was not applicable. However, we note that beside the initial cell selection, ring diameter analysis was fully automated, avoiding potential bias in the analysis.

Reporting for specific materials, systems and methods

We require information from authors about some types of materials, experimental systems and methods used in many studies. Here, indicate whether each material, system or method listed is relevant to your study. If you are not sure if a list item applies to your research, read the appropriate section before selecting a response.

Materials & experimental systems

n/a	Involved in the study
☒	☐ Antibodies
☒	☐ Eukaryotic cell lines
☒	☐ Palaeontology
☒	☐ Animals and other organisms
☒	☐ Human research participants
☒	☐ Clinical data

Methods

n/a	Involved in the study
☒	☐ ChIP-seq
☒	☐ Flow cytometry
☒	☐ MRI-based neuroimaging
